# Phototriggered Equilibrated and Transient Orthogonally Operating Constitutional Dynamic Networks Guiding Biocatalytic Cascades

**DOI:** 10.1021/jacs.3c13562

**Published:** 2024-02-29

**Authors:** Yu Ouyang, Itamar Willner

**Affiliations:** Institute of Chemistry, The Hebrew University of Jerusalem, Jerusalem 91904, Israel

## Abstract

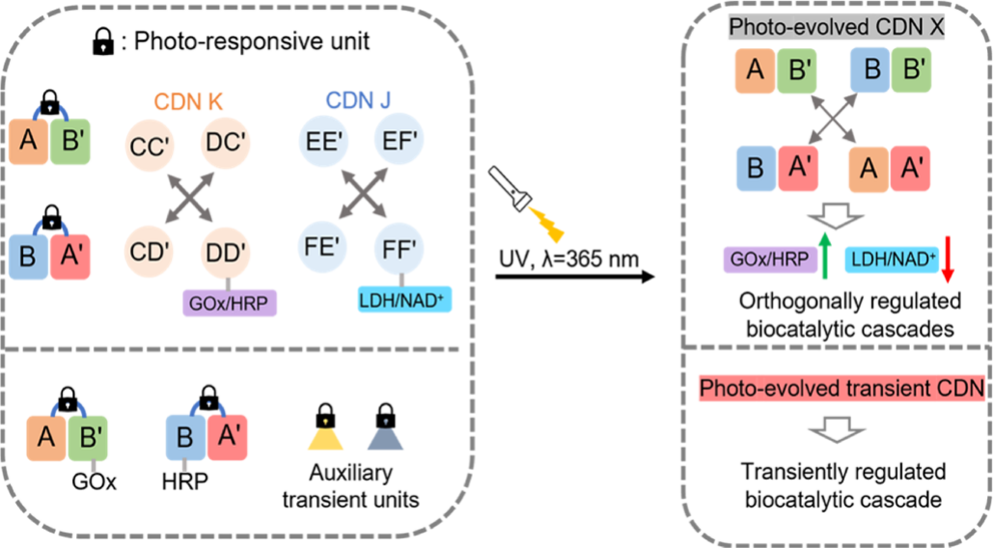

The photochemical deprotection of structurally engineered *o*-nitrobenzylphosphate-caged hairpin nucleic acids is introduced as a versatile method to evolve constitutional dynamic networks, CDNs. The photogenerated CDNs, in the presence of fuel strands, interact with auxiliary CDNs, resulting in their dynamically equilibrated reconfiguration. By modification of the constituents associated with the auxiliary CDNs with glucose oxidase (GOx)/horseradish peroxidase (HRP) or the lactate dehydrogenase (LDH)/nicotinamide adenine dinucleotide (NAD^+^) cofactor, the photogenerated CDN drives the orthogonal operation upregulated/downregulated operation of the GOx/HRP and LDH/NAD^+^ biocatalytic cascade in the conjugate mixture of auxiliary CDNs. Also, the photogenerated CDN was applied to control the reconfiguration of coupled CDNs, leading to upregulated/downregulated formation of the antithrombin aptamer units, resulting in the dictated inhibition of thrombin activity (fibrinogen coagulation). Moreover, a reaction module consisting of GOx/HRP-modified *o*-nitrobenzyl phosphate-caged DNA hairpins, photoresponsive caged auxiliary duplexes, and nickase leads upon irradiation to the emergence of a transient, dissipative CDN activating in the presence of two alternate auxiliary triggers, achieving transient operation of up- and downregulated GOx/HRP biocatalytic cascades.

## Introduction

Diverse biological processes, such as cell proliferation,^1,2^ motility,^3,4^ gene expression,^5−9^ and intracellular communication,^10,11^ are guided and regulated by complex dynamic networks and circuitries. These involve signal propagation and amplification,^12,13^ switching and oscillatory mechanisms,^14,15^ and programmed reaction patterns, revealing adaptive and hierarchically adaptive,^16,17^ cascaded,^18^ and spatiotemporal transient features.^19^ Inspired by nature, substantial research efforts are directed toward the development of artificial dynamic networks emulating the native circuits.^20−22^ The structural and functional information encoded in the base sequence of nucleic acids guides the stability of duplex nucleic acid structures^23,24^ and provides fundamental principles for duplex reconfiguration and displacement.^25^ Also, the base composition of nucleic acids guides the stabilization of supramolecular nucleic acid structures, such as triplex T-A·T or C-G·C^+^ or G-quadruplex structures.^26^ These strand-dictated structural motifs have been used to construct two-dimensional (2D) and three-dimensional (3D) DNA assemblies^27−29^ and as functional elements driving dynamic networks and circuitires.^30,31^ These include the assembly of DNA-based constitutional dynamic networks, CDNs, revealing adaptive,^32^ hierarchically adaptive,^33^ feedback-driven,^34^ and intercommunication functions.^35^ Diverse auxiliary triggers, such as fuel strands,^34,35^ G-quadruplexes,^36^ or light,^37^ were employed to induce signal-promoted structural reconfiguration of CDNs. Different applications of CDNs-involving control over material properties were demonstrated, e.g., switchable hydrogel properties^38^ and CDNs-guided operation of biocatalytic cascades.^39^ Moreover, spatiotemporal and transient operation of DNA-based circuitries attracts significant recent research efforts.^40−42^ Reaction modules driven by fuel strands in the presence of enzymes,^43−45^ DNAzymes, or light operated transient, dissipative, reaction circuits,^46^ leading to intermediate temporal formation of DNAzymes,^43^ temporal carriers of loads,^47^ temporal assembly of DNA structures, such as microtubules,^48^ or temporal aggregation of nanoparticles.^49^ In addition, nucleic acid-based circuitries acting as transcriptional oscillators,^50^ bistable transcriptional switches,^51^ transcriptional regulating networks,^17^ dynamic networks guiding orthogonal biocatalytic cascades,^52^ and transient DNA network-guided biocatalytic cascades^53^ were demonstrated.

Light is a particularly attractive trigger to switch the dynamic activities and reconfiguration of nucleic acid machines and structures, since it provides a rapid signal response and, often, does not require added chemical components, altering the composition of the reaction frameworks. Two general approaches were employed to trigger by light the reconfiguration of nucleic acid structures and their functions. One approach involved the application of photoisomerizable intercalator units modifying the nucleic acid strands, e.g., photoisomerizable *trans*/*cis* azobenzene units, controlling the stability of the DNA duplex and their light-induced duplex formation and separation.^54,55^ This approach was applied to design switchable DNA machines, such as “walkers,”^56,57^ tweezers,^58^ or mechanical rotaxanes,^59^ to control constitutional dynamic networks^37^ and to operate switchable transcription machinery.^60^ The second approach to control by light the reconfiguration of DNA structures and functions involves the caging of DNA structures by photoresponsive units, such as *o*-nitrobenzyl phosphate ester groups and their photochemical uncaging into functionally active DNA structures.^61^ Indeed, photoresponsive caged nucleic acid structures were applied for the light-induced activation of DNA machines, such as tweezers^62^ and walkers,^63^ operation of DNA machinery such as transcription circuits,^64^ CRISPR/Cas 9 machinery,^65^ and transient transformations such as light-induced ligation machinery employing caged adenosine triphosphate (ATP) and ligation templates^66^ or auxiliary photoresponsive acids modulating the dynamic pH-triggered formation and dissociation of DNA fibers.^67^

One of the challenges in developing synthetic dynamic circuitries involves, however, the precise engineering of the constituents comprising the networks, circuits, and reaction modules to prevent intracircuit perturbing cross-interactions while preserving the desired functions of the frameworks. While the advances in DNA nanotechnology defined basic rules and guidelines to formulate the duplex stabilities and programmed displacement efficacies,^25,68,69^ the search for developing new and efficient methods to assemble the circuits is still a challenge. In fact, the dynamic emergence and evolution of networks have attracted recent scientific efforts. Beyond providing basic principles to design the dynamic frameworks, the methods could introduce insights into the evolution of networks under prebiotic conditions.^70^ The fuel-triggered, enzyme-free reproduction and variation of CDNs from a pool of nucleic acid strand/nucleic acid hairpins generating DNAzyme units, leading to dynamic selection of CDNs, were realized.^71^ Alternatively, the enzyme-driven emergence of CDNs from caged constitutional frameworks was accomplished.^72^ Also, the enzyme-free catalytic hairpin assembly process was applied as a functional reaction module for the emergence and evolution of CDNs from a set of nucleic acid hairpins.^70^ Despite these advances, the development of additional scalable methodologies for the emergence and evolution of CDNs with enhanced complexities and functionalities is desirable.

Here, we wish to report on the light-induced evolution of a constitutional dynamic network (CDN) by photodeprotection of two *o*-nitrobenzyl phosphate-caged hairpin structures.^61,73^ The resulting CDN provides a reaction module for the triggered metabolic cleavage of hairpins, yielding fuel signaling strands for the activation of auxiliary CDNs operating in orthogonal biocatalytic cascades. Moreover, the concept of light-triggered evolution of CDNs is applied to engineer two *o*-nitrobenzyl phosphate-caged hairpins, yielding upon photodeprotection the emergence of a CDN guiding the directional fuel-triggered operation of auxiliary CDN circuits, leading to the upregulated/downregulated thrombin-stimulated catalyzed coagulation of fibrin to fibrinogen. Finally, a reaction module driving the phototriggered evolution of a CDN operating in the presence of two alternate fuels, an orthogonal upregulated/downregulated transient biocatalytic cascade, is introduced. The novel elements of the present study, as compared to previous art, and their contribution to the rapidly developing topic of dynamic DNA circuits include the following: (i) The light-triggered emergence of a functional CDN. (ii) The conjugation of the emerged CDN to auxiliary CDNs and the guided operation of orthogonal upregulated/downregulated biocatalytic cascades. (iii) The application of the light-triggered emergence of a CDN to assemble a dynamic network driving the guided upregulation/downregulation of the thrombin-biocatalyzed coagulation of fibrinogen. (iv) The light-induced evolution of a dissipative CDN driving the fueled orthogonally modulated transient operation of an upregulated/downregulated bienzyme cascade.

## Results and Discussion

**Figure 1:**
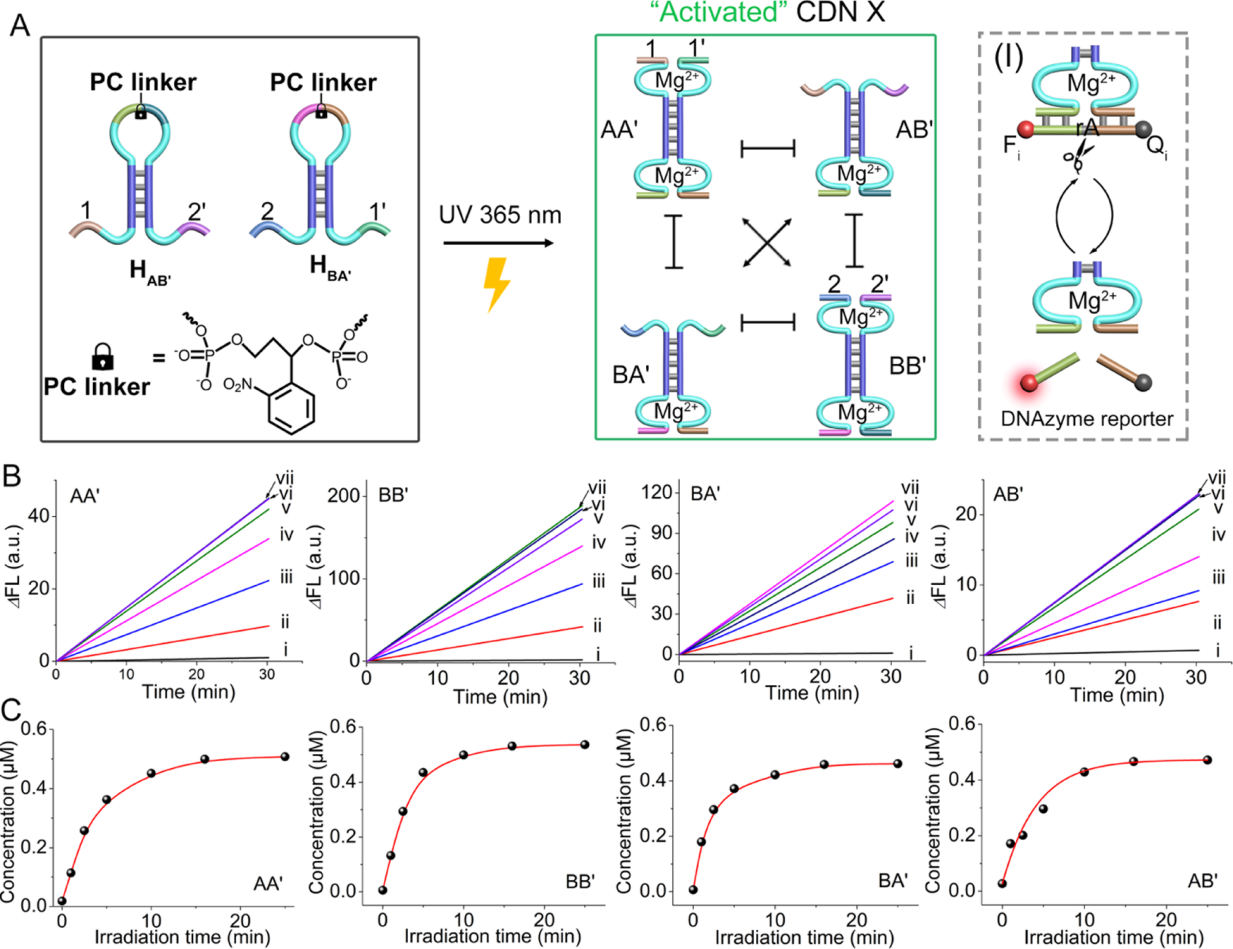
(A) Schematic light-triggered evolution of a constitutional dynamic network (CDN X) from a pair of photocleavable *o*-nitrobenzylphosphate-caged hairpin structures. Each of the evolved constituents includes a Mg^2+^-ion-dependent DNAzyme reporter unit transducing the content of the constituent by the rate of cleavage of the fluorophore/quencher-modified substrate associated with the DNAzyme reporter unit (Panel I-right). (B) Time-dependent fluorescence changes upon cleavage of the fluorophore/quencher-modified substrates associated with the constituents AA′, BB′, AB′, and BA′ in samples withdrawn from the reaction medium irradiated for different time intervals: (i) 0, (ii) 1, (iii) 3, (iv) 5, (v) 10, (vi) 15, and (vii) 25 min. (C) Concentrations of the constituents generated at different time intervals of irradiation of the reaction mixture of hairpins H_AB′_ and H_BA′_. The saturated equilibrated mixture of constituents is obtained after ca. 10 min of irradiation of the hairpins (λ = 365 nm).

Figure 1A depicts schematically the light-triggered emergence of a constitutional dynamic network, CDN X. Two hairpins H_AB_′ and H_BA_′ caged in their loop regions with *o*-nitrobenzyl phosphate photoresponsive units are photodeprotected at λ = 365 nm. The cleaved hairpin strands were engineered to include base complementarities that allow their re-equilibration into the [2 × 2] CDN X comprising four constituents AA′, AB′, BA′, and BB′. Each of the emerging constituents includes a Mg^2+^-ion-dependent DNAzyme unit. The DNAzyme units differ in the sequences comprising the binding arms of appropriate fluorophore/quencher-functionalized substrates, F*_i_*/Q*_i_*-S (S = ribonucleobase-modified DNA substrate). The Mg^2+^-ion-dependent DNAzymes act as reporter units, monitoring quantitatively the concentrations of the respective constituents. That is, by following the cleavage rates of the respective F*_i_*/Q*_i_*-S substrates associated with the constituents and using appropriate calibration curves, the equilibrated concentrations of the constituents are quantitatively evaluated. In addition to the Mg^2+^-ion-dependent DNAzyme reporter units, the parent photoresponsive hairpin structures were pre-engineered to include two additional DNAzyme units in constituents AA′ and BB′ of CDN X that are spatially positioned opposite to the DNAzyme reporter units and included binding arms (1/1′ and 2/2′). Figure 1B depicts time-dependent fluorescence changes corresponding to the cleavage of the F*_i_*/Q*_i_*-S substrates by the DNAzyme reporter units associated with the constituents of CDN X, generated upon photodeprotection of hairpins H_AB′_ and H_BA′_ for variable time intervals. The equilibrated concentrations of the constituents composing CDN X are controlled by the illumination time, applied to photodeprotect the caged hairpins. Figure 1C presents the concentrations of the constituents generated upon illumination (unlocking) of hairpins H_AB′_ and H_BA′_ for different time intervals (translation of the catalytic rates and applying the appropriate calibration curves shown in Figures S1 and S2). The concentrations of the constituents increase as the time interval for photodeprotection of the hairpins is prolonged. Note that the concentrations of the emerged constituents level off to a saturation level after ca. 10 min of photodeprotection.

**Figure 2:**
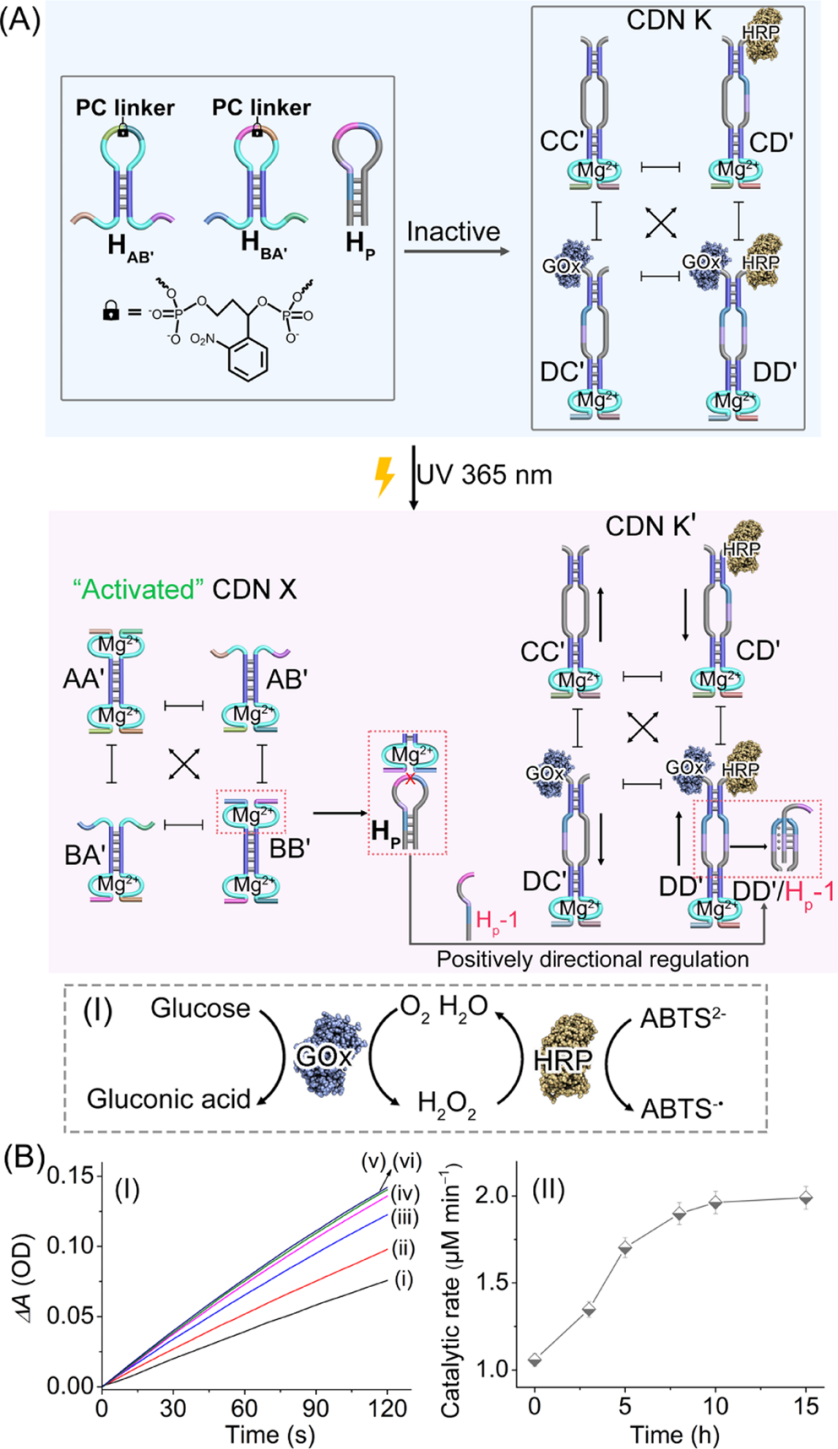
(A) Schematic light-triggered interaction between a reaction module consisting of photocleavable caged hairpins H_AB′_ and H_BA′_ and fuel hairpin H_P_ and an auxiliary CDN K operating the GOx/HRP biocatalytic cascade. Phototriggered cleavage of the reaction module evolves CDN X, which cleaves hairpin H_P_. The cleaved trigger H_P-1_ stabilizes constituent DD′ of auxiliary CDN K, resulting in upregulation of the GOx/HRP biocatalytic cascade (detailed in Panel I). (B) Panel I—time-dependent absorbance change generated by the GOx/HRP biocatalytic cascade (cf. A, Panel I) formed by samples withdrawn at time intervals from the CDN X/H_P-1_-triggered activation of CDN K (irradiation of the reaction module for 15 min): (i) 0, (ii) 3, (iii) 5, (iv) 8, (v) 10, and (vi) 15 h. Panel II shows temporal catalytic rates corresponding to the GOx/HRP cascade driven by the CDN X/H_P-1_-triggered activation of CDN K (derived from Panel I).

In the next step, the photochemically emerged CDN X was employed as a functional reaction module that activates auxiliary CDNs, guiding the temporal operation of biocatalytic cascades. The photogenerated CDN X is coupled to CDN K in which the biocatalytic cascade consisting of glucose oxidase (GOx) and horseradish peroxidase (HRP) is tethered to the respective constituents of CDN K. Alternatively, a second biocatalytic cascade consisting of lactate dehydrogenase (LDH) and the nicotinamide adenine dinucleotide (NAD^+^) cofactor was tethered to the constituents of CDN J (Figures 2A and S10A). The CDNs K and J carrying the different biocatalytic cascades interacted with caged hairpins H_AB′_ and H_BA′_ and the accompanying hairpins H_P_ or H_N_ to yield inactive coupled reaction modules. The photodeprotection of the reaction module consisting of hairpins H_AB′_, H_BA′_, and H_P_ leads, however, to the activation of CDN X that interacts and guides the control of CDN K and the associated GOx/HRP biocatalytic cascade (Figure 2A). The photochemical uncaging of hairpins H_AB′_ and H_BA′_ leads to the generation of CDN X comprising constituents AA′, AB′, BA′, and BB′. (The scheme corresponding to the enzyme-modified strand, the characterization of the 1:1 molar ratio of strand-to-enzyme, and the activity of the strand-modified enzymes *vs.* bare enzyme are displayed in Figures S3–S5.)

The catalytic cleavage of H_P_ by the Mg^2+^-ion-dependent unit associated with constituent BB′ (of CDN X) yields the trigger H_P-1_ that activates CDN K. The constituents composing CDN K include biloop domains. The biloop domain associated with constituent DD′ (in CDN K) was pre-engineered to interact with the trigger H_P-1_ to form a triplex structure, stabilizing constituent DD′. Stabilization of DD′ dynamically re-equilibrated CDN K by upregulating the content of DD′/H_P-1_, downregulating constituents CD′ and DC′, and the concomitant upregulation of constituent CC′. Upregulation of DD′/H_P-1_ increases the contents of the spatially proximate components of GOx and HRP, resulting in temporal enhancement of the GOx/HRP cascade. The biocatalytic cascade, Panel I, involves the aerobic oxidation of glucose to gluconic acid and H_2_O_2_ and the subsequent HRP-catalyzed oxidation of 2,2′-azino-bis(3-ethylbenzothiazoline-6-sulfonic acid), ABTS^2–^, to the colored product, ABTS^•–^ (λ = 420 nm). The temporal absorbance changes of ABTS^•–^ then provide a readout signal for the temporal CDN X-triggered activation of the CDN K-guided biocatalytic cascade. Evidently, the temporal performance of the biocatalytic GOx/HRP cascade is anticipated to be controlled by the time interval of illumination of hairpins H_AB′_ and H_BA′_ yielding the active constituent BB′ in CDN X, which catalyzes the generation of the intercommunicating trigger H_P-1_. The kinetics associated with the cleavage of H_P_ and the temporal supply of trigger H_P-1_ into CDN K dynamically controls the biocatalytic GOx/HRP cascade. Figure S6 depicts the time-dependent fluorescence change generated by the DNAzyme reporter units associated with the constituents of CDN K at different time intervals of CDN X-triggered reconfiguration of the CDN K → CDN K′, using CDN X produced by 15 min (Panel I) and 3 min (Panel II) of irradiation of the mixture of hairpins H_AB′_ and H_BA′_. Using the respective calibration curves of the reporter units, Figure S7, relating the cleavage rates of the fluorophore/quencher-modified substrates to the concentrations of the constituents, the temporal concentration changes of the constituents in CDN K → CDN K′ were evaluated, and these are presented in Figure S8. Evidently, the equilibration of the H_P-1_-triggered CDN K proceeds for a long time interval of ca. 10–15 h. The contents of constituents CC′ and DD′ are upregulated, whereas the contents of CD′ and DC′ are downregulated. The dynamic H_P-1_-triggered reconfiguration of CDN K is accompanied by the temporal enhancement of the GOx/HRP biocatalytic cascade. Figure 2B, Panel I, depicts the time-dependent absorbance changes of ABTS^•–^ formed by the GOx/HRP biocatalytic cascade associated with DD′/H_P-1_, generated in samples withdrawn at time intervals from CDN K activated by CDN X (CDN X was formed by irradiation of the reaction module consisting of H_AB′_ and H_BA′_ for 15 min). Knowing ε = 36,000 M^–1^ cm^–1^ (λ = 420 nm) for ABTS^•–^, the temporal catalytic rates for ABTS^•–^ formation were evaluated, and these are displayed in Figure 2B, Panel II. Evidently, the H_P-1_-triggered reconfiguration of CDN K → CDN K′ leads to temporal enhancement of the catalytic rates associated with the biocatalytic cascade that reached saturation values after 10 h. (For the results describing the CDN X/H_P-1_-activated GOx/HRP biocatalytic cascade in CDN K upon irradiation of the reaction module for a time interval of 3 min, see Figure S9.)

Similarly, the reaction module consisting of H_AB′_, H_BA′_, and H_N_, coupled to CDN J, comprising constituents EE′, EF′, FF′, and FE′, was subjected to photochemical deprotection of hairpins H_AB′_ and H_BA′_ yielding CDN X (Figure S10A). Formation of CDN X resulted in the cleavage of hairpin H_N_, and the cleaved fragmented strand intercommunicates CDN X with CDN J and guides the operation of the LDH/NAD^+^ biocatalytic cascade in CDN J. (The scheme corresponding to the synthesis of the F-modified LDH and F′-functionalized NAD^+^, characterization of their 1:1 molar ratio, and assessment of the activity of the nucleic acid-modified enzyme/cofactor are provided in Figures S3, S11, and S12). Trigger H_N-1_ is engineered to form a triplex structure with the biloop domain of constituent FE′ in CDN J, FE′/H_N-1_. Stabilization of constituent FE′ dynamically reconfigures CDN J to CDN J′, where FE′/H_N-1_ is upregulated, EE′ and FF′ are downregulated, and concomitantly constituent EF′ is upregulated. Upregulation of FE′/H_N-1_ decreases the content of constituent FF′ that is conjugated to the spatially proximate components of LDH and NAD^+^, resulting in the temporal decrease of the LDH/NAD^+^ biocatalytic cascade. The biocatalytic cascade corresponds to the LDH-catalyzed reduction of NAD^+^ by lactic acid, and the temporal reaction of NADH is probed by the interaction of NADH with the methylene blue (MB^+^) by following the absorbance changes of reduced MB^+^, MBH (λ = 664 nm), Figure S10A, Panel I. The temporal absorbance changes provide then a readout signal for the CDN X-induced activation of the CDN J-guided biocatalytic cascade. Accordingly, the temporal catalytic performance of the biocatalytic LDH/NAD^+^ cascade is anticipated to be controlled by the time interval of illumination of hairpins H_AB′_ and H_BA′_ generating the active constituent AA′ in CDN X, which catalyzes the generation of the intercommunicating H_N-1_ by the dynamic cleavage of H_N_, resulting in the temporal supply of trigger H_N-1_ dynamically affecting the LDH/NAD^+^ biocatalytic cascade.

The time-dependent fluorescence changes generated by the DNAzyme reporter units associated with CDN J at time intervals of the CDN X/H_N-1_-guided reconfiguration of CDN J→ CDN J′ are displayed in Figure S13. (CDN X is formed by 15 min of irradiation of the reaction module and deprotection of H_AB′_/H_BA′_.) Using appropriate calibration curves relating the rates of cleavage of the substrates by the DNAzyme reporters to the concentrations of the constituents in Figure S14, the temporal concentration changes of the constituents upon the CDN X/H_N-1_-induced reconfiguration of CDN J→ CDN J′ were evaluated, and these are displayed in Figure S15. Evidently, the reconfiguration of the CDN X/H_N-1_-triggered CDN J proceeds for a long time interval of ca. 10–15 h. The contents of the constituents (FE′ and EF′) are upregulated, whereas the contents of EE′ and FF′ are downregulated. The dynamic CDN X/H_N-1_-triggered reconfiguration of CDN J→ CDN J′ is accompanied by the temporal downregulation of the LDH/NAD^+^ biocatalytic cascade. The time-dependent absorbance changes of the LDH/NAD^+^ biocatalytic cascade operating at time intervals of the dynamically equilibrated CDN J triggered by H_N-1_ generated from photocleavable uncaging of H_AB′_ and H_BA′_ for a time interval of 15 min are displayed (Figure S10B, Panel I). Figure S10B, Panel II, shows the temporal catalytic rate of the LDH/NAD^+^ cascade associated with the reconfiguration of CDN J → CDN J′ generated by trigger H_N-1_ formed upon irradiation of the reaction module yielding CDN X for a time interval of 15 min. Obviously, the H_N-1_-triggered reconfiguration of CDN J → CDN J′ leads to inhibition of the biocatalytic cascade that reaches saturation values of the catalytic rate after 10 h. (For the results of H_N-1_-induced downregulation of the LDH/NAD^+^ biocatalytic cascade upon irradiation of the reaction module for a time interval of 3 min, see Figure S16.)

**Figure 3:**
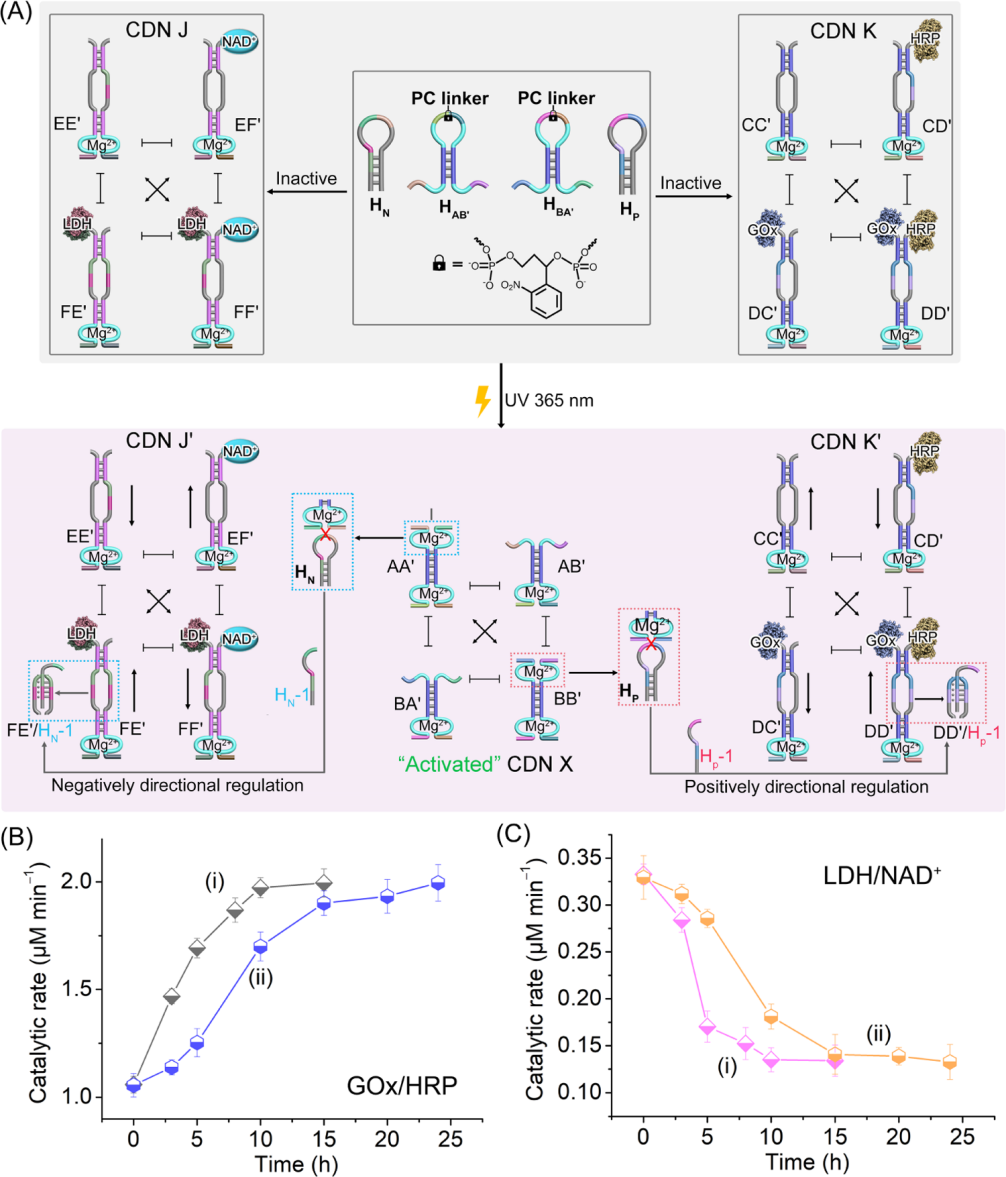
(A) Schematic application of a reaction module consisting of *o*-nitrobenzylphosphate-caged hairpins H_AB′_ and H_BA′_ and auxiliary hairpins H_P_ and H_N_ for the light-triggered activation of CDN X leading to the orthogonal concomitant CDN X/H_P-1_-triggered dynamic enhancement of the GOx/HRP cascade in CDN K and the CDN X/H_N-1_-triggered dynamic inhibition of the LDH/NAD^+^ cascade in CDN J. (B) Temporal catalytic rates of the GOx/HRP biocatalytic in reaction samples withdrawn from phototriggered CDN X/H_P-1_-triggered activation of CDN K, where curve (i): irradiation of the reaction module for 15 min and curve (ii): irradiation of the reaction module for 3 min, λ = 365 nm. (C) Temporal catalytic rates of the LDH/NAD^+^ biocatalytic in reaction samples withdrawn from phototriggered CDN X/H_N-1_-triggered activation of CDN J, where curve (i): irradiation of the reaction module for 15 min and curve (ii): irradiation of system for 3 min, λ = 365 nm.

In the next step, the light-induced emergence of CDN X was applied to guide the orthogonal operation of two biocatalytic cascades by applying a mixture of the CDN K-driven GOx/HRP and CDN J-driven LDH/NAD^+^ cascades. Figure 3A shows the reaction module consisting of the two inactive photoresponsive hairpins, H_AB′_ and H_BA′_, and two fuel hairpins H_P_ and H_N_, coupled to the two biocatalytic cascaded CDN K and CDN J. Irradiation of the module deprotects the hairpins, H_AB′_ and H_BA′_, and leads to the fast re-equilibration of CDN X. The resulting CDN X activates the concomitant catalytic cleavage of H_P_ and H_N_ by the DNAzymes associated with constituents BB′ and AA′ of CDN X to yield H_P-1_ and H_N-1_, respectively. The resulting H_P-1_ stabilizes constituent DD′ (DD′/H_P-1_) of CDN K, resulting in the temporal upregulation of constituents CC′ and DD′, and downregulation of constituents CD′ and DC′. The temporal upregulation of constituent DD′ leads to the temporal upregulation of the GOx/HRP biocatalytic cascade. Simultaneously, the resulting H_N-1_ stabilizes constituent FE′ in CDN J, leading to the upregulation of FE′ and EF′ and downregulation of EE′ and FF′ in CDN J. The downregulated content of FF′ results in the downregulation of the LDH/NAD^+^ cascade. That is, light-induced activation of the reaction module comprising H_AB′_ and H_BA′_ in the presence of the auxiliary CDN K and CDN J and triggers H_P_ and H_N_ leads to the orthogonal temporal upregulation of GOx/HRP and downregulation of LDH/NAD^+^ biocatalytic cascades, respectively. As the content of the constituents in CDN X is controlled by the time interval of the photochemical deprotection of the reaction module, the temporal orthogonal biocatalytic cascade driven by CDNs J and K is controlled by the primary photochemical step. Figure S17 presents the temporal concentration changes of the constituents in CDNs J and K stimulated by CDN X generated by photodeprotection of hairpins H_P_ and H_N_ in the reaction module upon photodeprotection for 15 and 3 min. The temporal concentration changes of constituents in CDNs J and K, stimulated by CDN X generated by different photochemical deprotection time intervals, guide the temporal orthogonal biocatalytic cascade proceeding in the system, as outlined in Figure 3B and 3C. The GOx/HRP biocatalytic cascade is temporally enhanced, while the LDH/NAD^+^ cascade is temporally inhibited.

**Figure 4:**
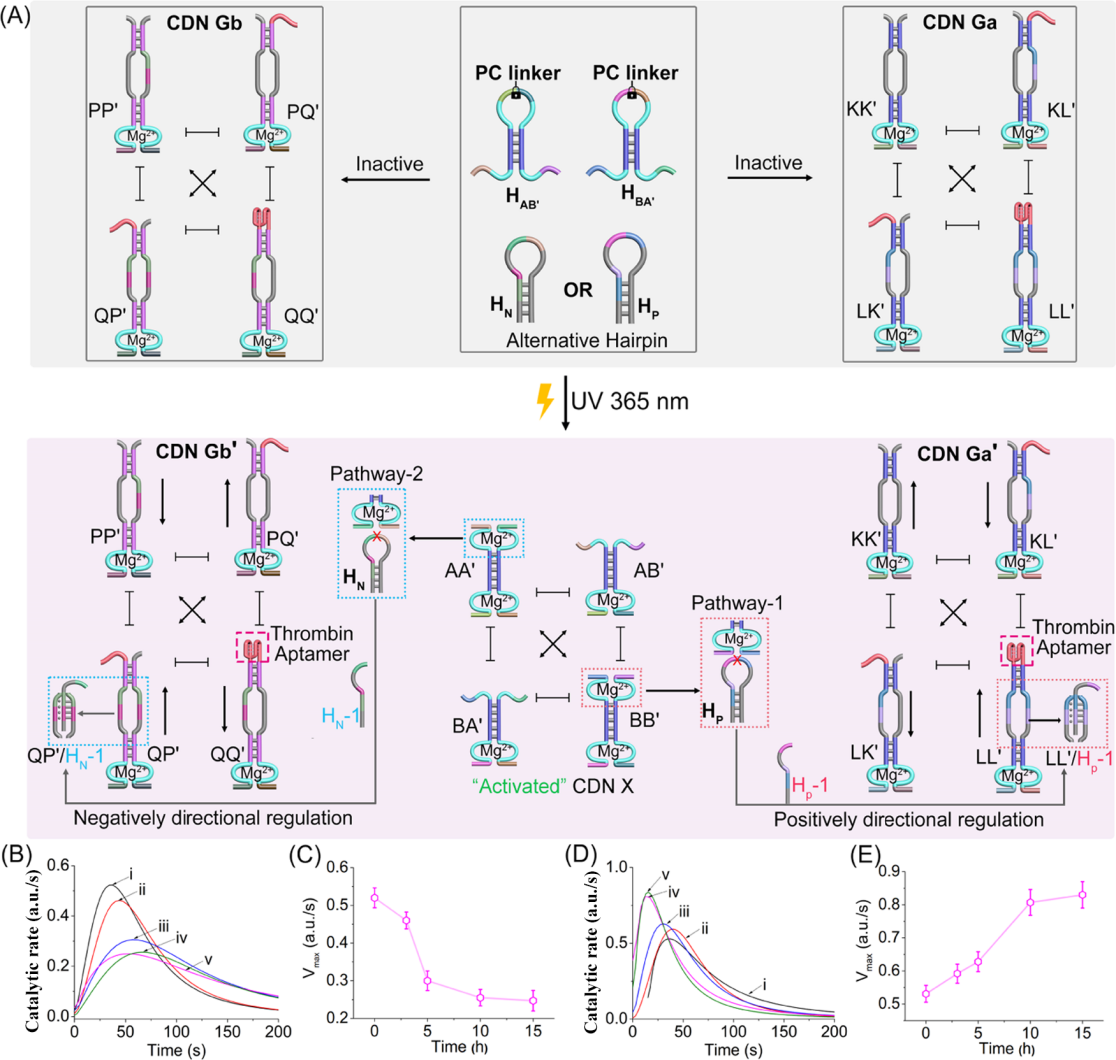
(A) Schematic reaction module consisting of *o*-nitrobenzylphosphate-caged hairpins H_AB′_ and H_BA′_ for the phototriggered evolution of CDN X and two auxiliary CDNs Ga and Gb. The alternate CDN X/H_P-1_-triggered CDN Ga leads to the inhibited thrombin coagulation activity (upregulation of constituent LL′) or the CDN X/H_N-1_-triggered CDN Gb leads to enhanced thrombin coagulation activity (downregulation of constituent QQ′). Note that photogenerated CDN X exists in the presence of the two CDNs Ga and Gb that are alternately activated by fuel hairpins H_P_ and H_N_. (B) Temporal rates of coagulation of fibrin to fibrinogen by reaction samples withdrawn at time intervals of operating the CDN X/H_P-1_-guided upregulation of LL′ in CDN Ga (inhibiting the coagulation process due to upregulation of LL′): (i) 0, (ii) 3, (iii) 5, (iv) 8, (v) 10, and (vi) 15 h. (C) Temporal maximum rates of coagulation of fibrin to fibrinogen stimulated by the CDN X/H_P-1_-triggered CDN Ga samples, shown in (B). (D) Temporal rates of coagulation of fibrin to fibrinogen by reaction samples withdrawn at time intervals of operating the CDN X/H_N-1_-guided downregulation of QQ′ in CDN Gb (enhancing the coagulation process due to downregulation of QQ′): (i) 0, (ii) 3, (iii) 5, (iv) 8, (v) 10, and (vi) 15 h. (E) Temporal maximum rates of coagulation of fibrin to fibrinogen stimulated by the CDN X/H_N-1_-triggered CDN Gb samples, shown in (D). Note that rates of coagulation correspond to the derivative of the transient light-scattering intensity curves corresponding to the reaction samples, cf. Figure S18.

The results presented so far demonstrated the light-induced deprotection of two hairpins forming a dynamically equilibrated [2 × 2] constitutional dynamic network guiding selective cleavage of auxiliary fuel hairpins that further interact with coupled CDN circuits. This concept was employed to construct a circuit of practical utility demonstrating the temporal upregulation/downregulation of thrombin activity toward blood clotting, as outlined in Figure 4A. The reaction module consists of two photoresponsive *o*-nitrobenzylphosphate-caged hairpins H_AB′_ and H_BA′_ and two alternative auxiliary hairpins H_P_ and H_N_. The reaction module is conjugated to two CDNs, namely, CDN Ga and CDN Gb. CDN Ga includes four constituents KK′, KL′, LK′, and LL′, where LL′ includes a tethered supramolecular antithrombin aptamer that is able to inhibit the catalytic activity of thrombin. CDN Gb includes four constituents PP′, PQ′, QP′ and QQ′, where constituent QQ′ is functionalized with the supramolecular antithrombin aptamer. Photodeprotection of hairpins H_AB′_ and H_BA′_ evolving the equilibrated CDN X results in the cleavage of hairpin H_P_, yielding trigger H_P-1_ that stabilizes the biloop structure of the constituent to yield LL′/H_P-1_. Stabilization of LL′ upregulates LL′ and KK′ and downregulates KL′ and LK′ constituents, yielding a CDN Ga → CDN Ga′ configuration with upregulated contents of the thrombin-inhibiting aptamer. Alternatively, subjecting the photodeprotected CDN X to hairpin H_N_ results in the cleavage of H_N_ by the DNAzyme units associated with constituent AA′, and the resulting H_N-1_ strand stabilizes constituent QP′ in CDN Gb. Stabilization of the constituent QP′ leads to the temporal upregulation of constituents QP′ and PQ′ and downregulation of PP′ and QQ′. The H_N-1_-guided downregulation of constituent QQ′ leads to a CDN framework with lower content of the supramolecular thrombin aptamer and, thus, to a framework of lower thrombin-inhibiting efficacy. That is, the parent reaction module in the presence of CDNs Ga and Gb reveals a base thrombin-inhibiting level controlled by the concentration of the supramolecular thrombin aptamer in the systems. The light-triggered activation of the reaction module in the presence of the alternative hairpin H_P_ or H_N_ leads to the directional up- or downregulation of the thrombin aptamer inhibition efficacy. Besides the temporal inhibition features of the circuit, it exhibits spatial functionalities reflected by the light-triggered operation of the system. Figure 4B demonstrates the directional temporal upregulated thrombin inhibition functions of the framework. The time-dependent light-scattering changes of the thrombin-induced coagulation of fibrin to fibrinogen upon the light-activated system in the presence of hairpin H_P_ are depicted in Figure S18A. At time *t* = 0, fast light-scattering intensities are observed, curve (i), indicating high coagulation. At longer time intervals of the CDN X-stimulated H_P-1_-triggered interaction with CDN Ga, where the CDN Gb is not affected, the time-dependent light-scattering intensity changes, curves (ii)–(v), turn slower, indicating slower fibrin-to-fibrinogen transitions and slower coagulation rates. The temporal catalytic rates corresponding to the thrombin-catalyzed coagulation rates (a derivative of the temporal light-scattering intensities shown in Figure S18A) are depicted in Figure 4B, and the temporal changes in the *V*_max_ values associated with the thrombin coagulation rates are displayed in Figure 4C. These results are consistent with the CDN X-induced downregulation of the thrombin activity upon the H_P-1_-triggered dynamic reconfiguration of CDN Ga to CDN Ga′. That is, at *t* = 0, the equilibrated concentration of LL′ at a high level leads to high base inhibition of thrombin and a slow thrombin coagulation rate. The H_P-1_-driven reconfiguration of CDN Ga upregulates the content of LL′, leading to enhanced inhibition of the thrombin-catalyzed coagulation. After a time interval of 10 h, the reconfiguration of the CDN Ga → CDN Ga′ reached the thermodynamically equilibrated level, resulting in the optimal level of the thrombin inhibition rate by the CDN Ga circuit, whereas CDN Gb remains an inactive state. Similarly, Figure S18B shows the time-dependent light-scattering intensities upon treatment of the reaction module coupled with CDN Ga and CDN Gb with the photogenerated CDN X in the presence of hairpin H_N_. At time *t* = 0, the time-dependent light-scattering intensity changes of the thrombin-catalyzed fibrin to fibrinogen are relatively slow, curve (i), reflecting low coagulation rates. As the time of interaction with H_N-1_ is prolonged, the time-dependent light-scattering changes are faster, curves (ii)–(v), indicating higher coagulation rates and lower inhibition efficacies of the antithrombin aptamer in reconfigured CDN Gb (constituent, QQ′). Figure 4D shows temporal catalytic rates of thrombin-catalyzed coagulation of fibrin, curves (i)–(v), and Figure 4E depicts the temporal *V*_max_ values for coagulation of fibrin upon the dynamic H_N-1_-guided reconfiguration of CDN Gb to Gb′. The coagulation rates increase as the reconfiguration process proceeds, reaching a saturation value after ca. 10 h that corresponds to the time interval required to thermodynamically equilibrate CDN Gb → Gb′. H_N-1_-reconfigured CDN Gb → CDN Gb′ leads to the temporal dynamic downregulation of the content of constituent QQ′ that guides the enhancing efficacy of the thrombin aptamer toward thrombin. That is, the phototriggered generated CDN X leads, in the presence of CDNs Ga and Gb and alternate hairpins H_P_ or H_N_, to downregulated/upregulated fibrin coagulation rates by the dynamic light-responsive reaction module.

**Figure 5:**
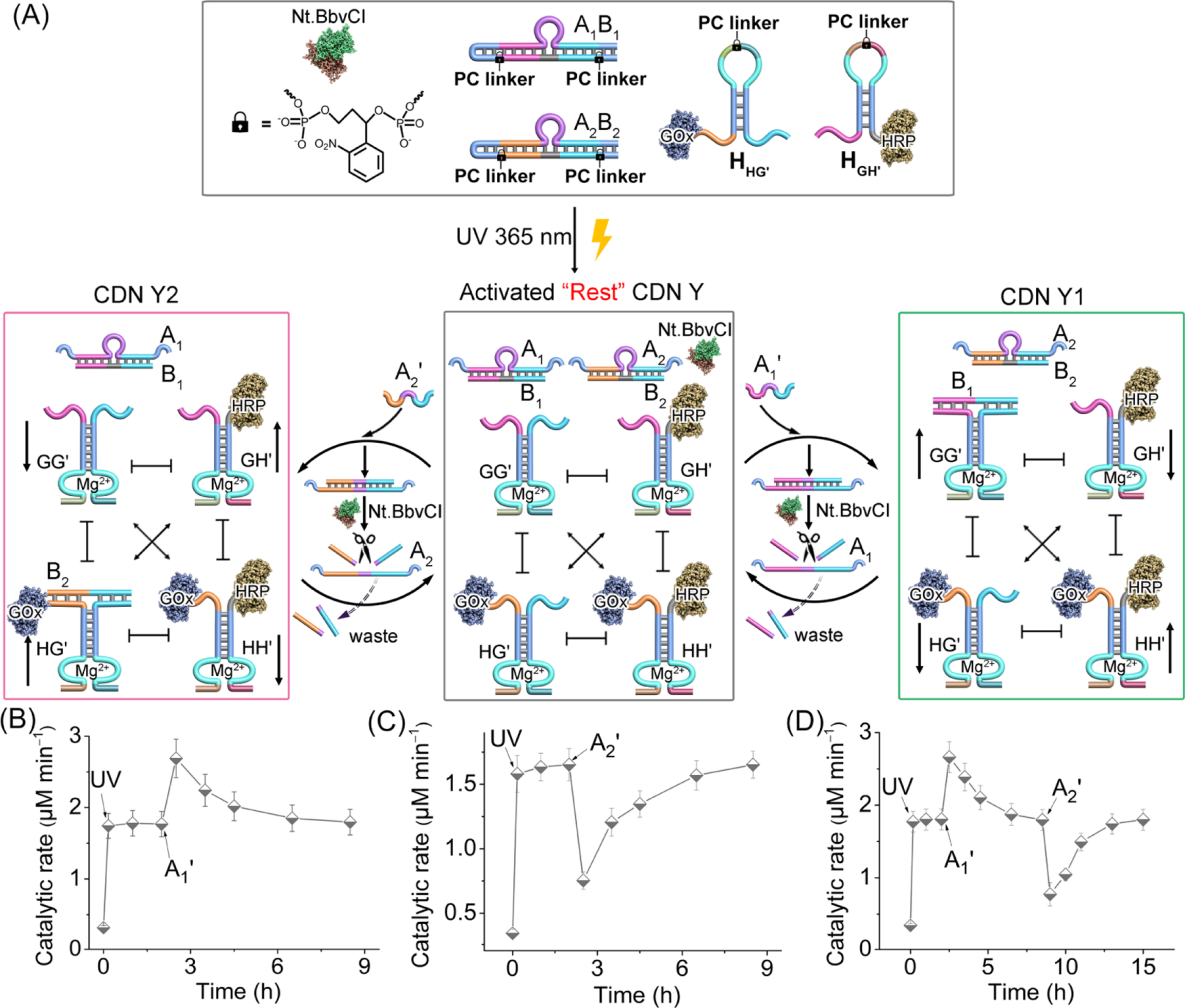
(A) Schematic reaction module for the phototriggered activation of a CDN Y guiding the fueled transient reconfiguration into CDN Y1 or CDN Y2 driving transient upregulated/downregulated biocatalytic cascades. (B) Temporal catalytic rates corresponding to the biocatalytic GOx/HRP cascade operating in samples of the A_1_′-fueled transient reconfiguration of light-emerged dissipative CDN Y to Y1 and back. The light-triggered activation of the reaction module (15 min irradiation, followed by 10 min of equilibration of CDN Y) is accompanied by the A_1_′-fueled reconfiguration of CDN Y to CDN Y1 driving the transient upregulated GOx/HRP biocatalytic cascade. (C) Temporal catalytic rates corresponding to the biocatalytic GOx/HRP cascade operating in samples of the A_2_′-fueled transient reconfiguration of light-emerged dissipative CDN Y to Y2 and back. The light-triggered activation of the reaction module (15 min irradiation, followed by 10 min of equilibration of CDN Y) is accompanied by the A_2_′-fueled reconfiguration of CDN Y to CDN Y2 driving the transient downregulated GOx/HRP biocatalytic cascade. (D) Cyclic temporal catalytic rates corresponding to the GOx/HRP biocatalytic cascade operating in samples of sequential A_1_′- and A_2_′-fueled transient reconfiguration in the reaction module.

The light-triggered evolution of an orthogonal constitutional dynamic network operating catalytic cascades was further applied to develop light-triggered constitutional dynamic networks driving transient orthogonal biocatalytic cascades (Figure 5). The reaction module consists of photoresponsive *o*-nitrobenzylphosphate-caged GOx-modified hairpin H_H*G*′_ and HRP-functionalized hairpin H_GH′_. (For the characterization of the enzyme-DNA conjugates, see Figures S19 and S20.) Two photoresponsive *o*-nitrobenzylphosphate-bridged duplex hairpins A_1_B_1_ and A_2_B_2_ are added to the reaction module as constituents together with the nicking enzyme Nt.BbvCI, yielding a biocatalytically inactive dissipative reaction framework. Photoirradiation of the reaction module (λ = 365 nm) deprotects all *o*-nitrobenzylphosphate bridging units, resulting in dynamically equilibrated CDN Y accompanied by uncaged duplexes A_1_B_1_ and A_2_B_2_ as a “rest” functional framework. The spatial proximity between GOx and HRP in constituent HH′ allows, in the presence of glucose, the aerobic oxidation of glucose and the operation of the GOx/HRP biocatalytic cascade. The constituents in CDN Y are conjugated to Mg^2+^-ion-dependent DNAzymes, acting as reporter units that allow quantitative assessment of the concentrations of the equilibrated constituents by following the cleavage rates of the fluorophore/quencher-modified substrates. Subjecting the “Rest” CDN Y to the auxiliary fuel strand, A_1_′, results in the displacement of duplex A_1_/B_1_ to yield A_1_/A_1_′ and the stabilization of constituent G*G*′ by released B_1_ and the reconfiguration of CDN Y to CDN Y1. Stabilization of G*G*′ leads to upregulation of G*G*′, the concomitant upregulation of HH′, and the downregulation of GH′ and H*G*′. The upregulation of HH′ results in the enhancement of the GOx/HRP biocatalytic cascade beyond the parent biocatalytic activity in CDN Y. Strand A_1_′ in duplex A_1_/A_1_′ was engineered, however, to be nicked by the nicking enzyme, NtBbvCI, to yield fragmented “waste” products. The released strand A_1_ displaces B_1_, destabilizing constituent G*G*′, resulting in the temporal recovery of CDN Y1 as the parent CDN Y, demonstrating the original GOx/HRP cascade biocatalytic activity characteristic to CDN Y. Similarly, subjecting the light-induced transient reaction module to fuel strand A_2_′ results in the displacement of duplex A_2_/B_2_, leading to the stabilization of constituent H*G*′ and to reconfiguration of CDN Y to CDN Y2 with the concomitant formation of duplex A_2_/A_2_′. The stabilization of H*G*′ leads to the upregulation of constituent H*G*′ and GH′ and the downregulation of G*G*′ and HH′. The downregulation of HH′ leads to a decrease in the rate of the biocatalytic GOx/HRP cascade in CDN Y2, as compared to the parent GOx/HRP biocatalytic cascade in CDN Y. The A_2_′ strand in duplex A_2_/A_2_′ is, however, designed to include a nicking site for Nt.BbvCI, resulting in the cleavage of A_2_′ and the release of fragmented waste and free A_2_. The released A_2_ displaces B_2_, leading to the temporal reconfiguration of CDN Y2 to CDN Y, resulting in the higher original level of the GOx/HRP biocatalytic cascade associated with CDN Y. That is, the reaction module shown in Figure 5A leads to the orthogonal A_1_′/A_2_′-fueled transient cascaded GOx/HRP biocatalytic process in photogenerated CDN Y.

The temporal transient concentration changes of the constituents upon the A_1_′-fueled reconfiguration of CDN Y → Y1 → Y and dynamic A_2_′-fueled transitions of CDN Y → Y2 → Y are transduced by the Mg^2+^-ion-DNAzyme reporter units. Figure S21 presents the temporal cleavage rates of the fluorophore/quencher-modified substrates associated with the DNAzyme units upon the A_1_′-fueled transition of CDN Y → Y1 → Y. Using the appropriate calibration curves, Figure S22, the temporal, transient, and concentration changes of the constituents of light-generated CDN Y, were evaluated, and these are displayed in Figure S23. Evidently, the constituents G*G*′ and HH′ reveal a temporal transient upregulation followed by a transient recovery to the base level characterizing CDN Y, and constituents GH′ and H*G*′ are temporally downregulated, followed by a transient recovery to the base concentration level characterizing equilibrated CDN Y. Similarly, Figure S24 presents the temporal cleavage rates of the substrates by the DNAzyme units associated with CDN Y upon the A_2_′-fueled transition of CDN Y → Y2 → Y. Using the appropriate calibration curves, the temporal, transient, constituent concentration changes of the light-generated CDN Y were evaluated, and these are displayed in Figure S25. That is, constituents GH′ and H*G*′ reveal a temporal upregulation followed by a transient recovery to the base level characterizing CDN Y, and constituents G*G*′ and HH′ are temporally downregulated followed by a transient recovery to the base concentration level characterizing equilibrated CDN Y. Moreover, the A_1_′- and A_2_′-fueled transient reconfiguration of CDN Y into CDN Y1 or CDN Y2 could be applied in a cyclic consecutive order controlled by the fuel strands. This is exemplified in Figure S26 using a two-cycle reconfiguration of CDN Y, applying consecutively fuel strands A_1_′ and A_2_′ to complete the transient transitions of CDN Y → Y1 → Y→Y2 → Y.

Realizing transient control over the compositions of the constituents of the photogenerated reaction module by means of fuel strand A_1_′ or A_2_′, we applied the dynamic behavior of the network to dynamically dictate the transient biocatalytic cascades accompanying the networks. The time-dependent absorbance changes of ABTS^•–^ generated by the GOx/HRP cascade by samples of the system at the time interval of transition CDN Y → Y1 → Y are displayed in Figure S27A. The respective temporal catalytic rates of the biocatalytic cascade are displayed in Figure 5B. Evidently, the A_1_′-fueled transition of light-generated CDN Y to Y1 is accompanied by an increase in the catalytic rate of the GOx/HRP cascade followed by a temporal decrease in the catalytic rates of the system that reaches the parent catalytic rate of the biocatalytic cascade characterizing CDN Y, after a time interval of ca. 7 h. These transient behaviors of the GOx/HRP biocatalytic cascade are consistent with the A_1_′-fueled transient concentration changes of constituent HH′ in CDN Y upon the transition of CDN Y → Y1 → Y. Similarly, Figure S27B depicts the time-dependent absorbance changes of ABTS^•–^ of samples of the system undergoing A_2_′-fueled transitions of CDN Y → Y2 → Y. Figure 5C depicts the catalytic rates of the GOx/HRP biocatalytic cascade upon the transient transformation CDN Y → Y2 → Y. Evidently, the A_2_′-fueled transient reconfiguration of CDN Y to CDN Y2 and back to CDN Y is accompanied by rapid inhibition of the GOx/HRP cascade, followed by a transient recovery of the parent catalytic rate of the biocatalytic cascade characterizing CDN Y, after a time interval of ca. 7 h. Figure 5D depicts the sequential transient up- and downregulation of the biocatalytic cascade upon applying sequentially fuel strands A_1_′ and A_2_′ on the photogenerated CDN Y.

## Conclusions

The photochemical deprotection of structurally engineered *o*-nitrobenzylphosphate-caged nucleic acid hairpin structure was introduced as a versatile method to evolve constitutional dynamic networks, CDNs. The evolved CDNs were used as functional reaction modules to orthogonally reconfigure two auxiliary CDNs, in the presence of fuel hairpins. By chemical modification of the constituents of the auxiliary CDNs with biocatalytic components, the orthogonal operation of biocatalytic cascades, guided by the dynamically reconfigured CDNs, was demonstrated. Moreover, the photochemically evolved CDN was coupled to two auxiliary CDNs guiding the upregulated/downregulated formation of the antithrombin aptamer, resulting in the dictated inhibition of thrombin (fibrinogen coagulation). In addition, a reaction module consisting of two *o*-nitrobenzylphosphate-caged DNA hairpins modified with glucose oxidase (GOx) and horseradish peroxidase (HRP) and two auxiliary *o*-nitrobenzylphosphate-caged hairpin duplexes, in the presence of nickase, was used as a functional framework for the phototriggered assembly of a CDN network revealing temporally controlled transient biocatalytic functions. Photochemical deprotection of the reaction module results in the evolution of a functional CDN assembly that in the presence of two alternative fuel strands leads to an upregulated or downregulated transient GOx/HRP biocatalytic cascade. Besides the phototriggered evolution of networks guiding orthogonal biocatalytic cascades or transient upregulated/downregulated temporally operating biocatalytic cascades, the phototriggered evolution of networks has a substantially broader impact. By photodeprotection of three or four *o*-nitrobenzylphosphate-caged DNA hairpins, networks of higher dimensionalities for hierarchical dynamic control may be envisaged. Furthermore, one of the challenges in the future application of dynamic networks involves their integration in a cellular environment or synthetic protocell assemblies. While the integration of multicomponent constituents in a precise dose- and concentration-controlled manner is impossible, the incorporation of a limited number of photoresponsive caged constituents, or even a single oligomer photoresponsive structure, could resolve this problem. The spatiotemporal photodeprotection of the caged components could lead to the self-assembly of the networks in the desired reaction volumes. Moreover, the photodeprotection of caged hairpin DNA structures and their dynamic reconfiguration could be of broad applicability in materials science, e.g., the preparation of stimuli-responsive DNA hydrogels, DNA-gated carriers, and more.

## Supporting Information Available

Experimental section; sequences used in this study; detailed preparations of single modified conjugates of nucleic acid/enzyme or cofactor; materials; characterization; methods; calibration curves; and additional results (PDF)
