# Supplementary material for: Phototriggered Equilibrated and Transient Orthogonally Operating Constitutional Dynamic Networks Guiding Biocatalytic Cascades
Source: J Am Chem Soc. 2024 Feb 29;146(10):6806–16. doi: 10.1021/jacs.3c13562 (PMC10941189; doi:10.1021/jacs.3c13562)
Supplement: Supplementary file 1 — ja3c13562_si_001.pdf [file ja3c13562_si_001.pdf]

Supporting Information

Phototriggered Equilibrated and Transient Orthogonally Operating Constitutional Dynamic Networks Guiding Biocatalytic Cascades

Yu Ouyang, Itamar Willner*

Institute of Chemistry, The Hebrew University of Jerusalem, Jerusalem 91904, Israel

*Email: itamar.willner@mail.huji.ac.il

Materials and Methods

Materials

Succinimidyl 3-(2-pyridyldithio)propionate (SPDP), 4-carboxyphenylboronic acid, 1-ethyl-3-(3-dimethylaminopropyl)carbodiimide (EDC), N-hydroxysulfosuccinimide (NHS), Glucose oxidase from *Aspergillus niger* (GOx, 187300 U g^{-1}), peroxidase from horseradish, type II (HRP, 219000 U g^{-1}), nicotinamide adenine dinucleotide (NAD^+), lactate dehydrogenase from *rabbit muscle* (LDH, 550 U mg^{-1}), pyruvic acid, glucose, 2,2′-azino-bis(3-ethylbenzothiazoline-6-sulfonic acid) ($ABTS^{2-}$), methylene blue (MB^+), hydrazine hydrate, and lactic acid were purchased from Sigma-Aldrich. rCutSmart Buffer was purchased from New England BioLabs Inc.. DNA oligonucleotides were purchased from Integrated DNA Technologies Inc.

Instrumentation

Absorption spectra were recorded at 25 °C using a UV-2450 spectrophotometer (Shimadzu), a cuvette of 50 µL volume (made of quartz suprasil, Hellma Analytics) was used in these experiments. Fluorescence spectra were recorded at 25 °C using a Cary Eclipse Fluorometer (Varian Inc), a cuvette of 100 µL volume (disposable cuvette, Brand GMBH, Wehrheim, Germany) was used in these experiments. The enzyme/cofactor-DNA conjugates purification were implemented by anion-exchange fast protein liquid chromatography (FPLC) with MonoQ HR 5/5 column (GE Healthcare, 5 × 50 mm, 1 mL bearing sample volume). UV light source is carried on THORLABS device including LED (M365LP1-C2) and driver (M00760962) with 3 mW/cm^2.

Summary of nucleic acid strands in this study

$H_{AB'}$	5′-CAACTCAGCGATCCAATTACTAACCACCCATGTTCGTCA /iSpPC/ GATATCAGCGATGTTAGTAATTGG CACCACCCATGTTGAGTG-3′
$H_{BA'}$	5′-GTAGTCAGCGATCCAATTACTAACCACCCATGTTACTCT/iSpPC/ CTGCTCAGCGATGTTAGTAATTGGCACCCATGTTCTGTC-3′
C	5′-GTCCTCAGCGATCTCAAATTGACTTATTATCTTTCCTTTTCCGTTAAACAC-3′
C′	5′-GTGTTTAACCTTTATTTATTTATTTATTTCCAATTTGAG CACCCATGTTTCAGT-3’
D	5′-CTGTTCAGCGATCTCAAATTGACT*TCTCTTCTTT*ATTTATTAGTTAAACACTTT-SH-3′
D′	5′-SH-TTTGTGTTTAACCATTTTCC*TTTCTTCTCT*TACCAATTTGAG CACCCATGTTCCTGA-3′
H_P	5′-ATGCAAAGAAGAGACATAGACAGATrAGGAGTTGAAACAATCTTCTTTGCATA-3′
E	5′-CCATTCAGCGATCAGTAAACACTTTATTTAA*TTTCTCCTTT* CCACAAATGACTTT -3′
E′	5′-NH_2-TTTGTCATTTGTAA*TTTCCTCTTT*TCTTCCTTAATGTTTACTG CACCCATGTTTAGCT-3′
F	5′-GTCCTCAGCGATCAGTAAACACCTTCCTTCTTTTATTTATTTAACAAATGAC-SH-3′
F′	5′-NH_2-GTCATTTGTATTTATTTATTTATTTATTTAATGTTTACTGCACCCATGTTACTCT-3′

H_N	5′-AGCAAAGGAGAAACATACACTCATrAGGACTACAAACAATCTCCTTTGCTAT-3′
K	5′-GTCCTCAGCGATCTCAAATTGACTTATTATCTTTCCTTTTCCGTTAAACAC -3′
K′	5′-GTGTTTAACCTTTATTTATTTATTTATTTCCAATTTGAG CACCCATGTTTCAGT-3′
L	5′-CTGTTCAGCGATCTCAAATTGACT*TCTCTTCTTT*ATTTATTAGTTAAACACT TGGTTGG-3′
L′	5′-GGTTGGTG *T* GTGTTTAACCATTTTCC*TTTCTTCTCT*TACCAATTTGAGCACCCATGTTCCTGA-3′
Q	5′-CCATTCAGCGATCAGTAAACACTTTATTTAA*TTTCTCCTTT*CCACAAATGAC TGGTTGG-3′
Q′	5′-*GGTTGGTG* GTCATTTGTATTTATTTATTTATTTATTTAATGTTTACTGCACCCATGTTACTCT-3′
P	5′-GTCCTCAGCGATCAGTAAACACCTTCCTTCTTTTATTTATTTAACAAATGAC-3′
P′	5′-TTTGTCATTTGTAA*TTTCCTCTTT*TCTTCCTTAATGTTTACTG CACCCATGTTTAGCT-3′
$H_{HG'}$	5′-ACAGAAGAACCGTAAGTATTTTG CACCCATGTTACTCT /PC/ CTGCTCAGCGATCAAAATACTTACCCCATCACAAA AATTT-SH-3′
$H_{GH'}$	5′-SH-AAGTAAGTATTTTGCACCCATGTTCGTCA/PC/ GATATCAGCGATCAAAATACTTAC AGACACAACAA-3′
A_1B_1	5′-CAGGCACAGAAGAACGCTGAGGGACACAACAACGTCTTTGA/PC/ CGTTGTTGTGTCACGTTCTTCTGTG /PC/ CCTG-3′
A_2B_2	5′-CAGGGACAGAAGAACGCTGAGGCCATCACAAACCTTTCTAAG/PC/ AAAGGTTTGTGATGGACGTTCT TCTGTC /PC/CCTG-3′
$A_1′$	5′-GTGTCCCTCAGCGTT-3′;
$A_2′$	5′-GATGGCCTCAGCGTT-3′
F_{cali}	5′-Cy5-TCGTCCTCAGCT-3′
Q_{cali}	5′-AGCTGAGGACGA-BHQ2-3′
Substrates	sub1 (AA′): 5′-FAM-AGAGTATrAGGATATC-BHQ1-3′<br>sub2 (BB′): 5′-ROX-TGACGATrAGGAGCAG-BHQ2-3′<br>sub3 (BA′): 5′-Cy5-AGAGTATrAGGAGCAG-BHQ2-3′<br>sub4 (AB′): 5′-FAM-TGACGATrAGGATATC-BHQ1-3′<br>sub5 (DC′): 5′-FAM-ACTGAATrAGGAACAG-BHQ1-3′<br>sub6 (CD′): 5′-ROX-TCAGGATrAGGAGGAC-BHQ2-3′<br>sub7 (CC′): 5′-Cy5-ACTGAATrAGGAGGAC-BHQ2-3′<br>sub8 (DD′): 5′-FAM-TCAGGATrAGGAACAG-BHQ1-3′<br>sub9 (EE′): 5′-FAM-AGCTAATrAGGAATGG-BHQ1-3′<br>sub10 (FF′): 5′-ROX- AGAGTATrAGGAGGAC-BHQ2-3′<br>sub11 (FE′): 5′-FAM-AGCTAATrAGGAGGAC-BHQ1-3′<br>sub12 (EF′): 5′-FAM-AGAGTATrAGGAATGG-BHQ1-3′<br>sub1-noFQ (AA′): 5′-AGAGTATrAGGATATC-3′<br>sub2-noFQ (BB′): 5′-TGACGATrAGGAGCAG-3′<br>sub3-noFQ (BA′): 5′-AGAGTATrAGGAGCAG-3′<br>sub4-noFQ (AB′): 5′-TGACGATrAGGATATC-3′<br>sub5-noFQ (DC′): 5′-ACTGAATrAGGAACAG-3′<br>sub6-noFQ (CD′): 5′-TCAGGATrAGGAGGAC-3′<br>sub7-noFQ (CC′): 5′-ACTGAATrAGGAGGAC-3′<br>sub8-noFQ (DD′): 5′-TCAGGATrAGGAACAG-3′<br>sub9-noFQ (EE′): 5′-AGCTAATrAGGAATGG-3′<br>sub10-noFQ (FF′): 5′-AGAGTATrAGGAGGAC-3′<br>sub11-noFQ (FE′): 5′-AGCTAATrAGGAGGAC-3′<br>sub12-noFQ (EF′): 5′-AGAGTATrAGGAATGG-3′
Note	Arm of the Respective DNAzyme make with underline; the sequences of triplex maked as italic type; subpart of antithrombin aptamer maked as green color; duplex parts of constituents of respective CDNs maked as blue and pink color.

Methods

Modification of strand D or H with GOx

GOx (80 µl, 100 µM) was subjected to SPDP (2.4 µL, 0.01 M) in HEPES buffer (10 mM, pH = 7.2) for 1 h. Excess SPDP was removed by washing three times with Amicon 10 kDa cutoff filters. Next, SPDP-modified GOx was conjugated to strand D or H (eightfold excess) through a disulfide bond exchange of the activated pyridyldithiol group. The reaction mixture was stirred in HEPES buffer (10 mM, pH = 7.2) for 2 h. The coupling efficiency was evaluated by monitoring the increase in absorbance at 343 nm due to the release of pyridine-2-thione (extinction coefficient, 8,080 M^{-1} cm^{-1}). Finally, the excess DNA was removed by washing with Amicon 30 kDa cutoff filters. The DNA labelling ratio of the purified enzyme-DNA conjugates was also quantified by measuring the absorbance ratio at 260 and 280 nm. The enzymatic activity of the DNA-modified GOx was ~70% of the activity of the native enzyme.

Modification of strand D′ or H′ with HRP

HRP (80 µl, 100 µM) was subjected to SPDP (5.6 µL, 0.01 M) in HEPES buffer (10 mM, pH = 7.2) for 1 h. Excess SPDP was removed by washing three times with Amicon 10 kDa cutoff filters. Next, SPDP-modified HRP was conjugated to strand D′ or H′ (eightfold excess) through a disulfide bond exchange of the activated pyridyldithiol group. The reaction mixture was stirred in HEPES buffer (10 mM, pH = 7.2) for 2 h. The coupling efficiency was evaluated by monitoring the increase in absorbance at 343 nm due to the release of pyridine-2-thione (extinction coefficient, 8,080 M^{-1} cm^{-1}). Finally, the excess DNA was removed by washing with Amicon 30 kDa cutoff filters. The DNA labelling ratio of the purified enzyme-DNA conjugates can also be quantified by measuring the absorbance ratio at 260 and 280 nm. The enzymatic activity of the DNA-modified HRP was ~73% of the activity of the native enzyme.

Modification of strand F with LDH

LDH (100 µl, 23 µM) was subjected to SPDP (4 µL, 0.5 M) in HEPES buffer (10 mM, pH = 8.0) for 1 h. Excess SPDP was removed by washing three times with Amicon 10 kDa cutoff filters. Next, SPDP-modified LDH was conjugated to strand F (eightfold excess) through a disulfide bond exchange of the activated pyridyldithiol group. The reaction mixture was stirred in HEPES buffer (10 mM, pH = 7.2) for 2 h. The coupling efficiency was evaluated by monitoring the increase in absorbance at 343 nm due to the release of pyridine-2-thione (extinction coefficient, 8,080 M^{-1} cm^{-1}). Finally, the excess DNA was removed by washing with Amicon 30 kDa cutoff filters. The DNA labelling ratio of the purified enzyme-DNA conjugates can also be quantified by measuring the absorbance ratio at 260 and 280 nm. The enzymatic activity of the DNA-modified LDH was ~67% of the activity of the native enzyme.

Modification of strand F′ with NAD^+

4-carboxyphenylboronic acid (2 µL, 50 mM) was subjected to EDC (3 µL, 10 mg mL^{-1}) in 200 µL of MES buffer (10 mM, pH = 5.5) for 5 min. Subsequently, NHS (5 µL, 10 mg mL^{-1}) in 200 µL of PBS buffer (50 mM, pH = 7.2) was added and the mixture shaken for 10 min. Strand F′ (10 µL, 1 mM) was then added and the mixture shaken for

2 ~ 3 h. Excess reagents were removed by washing three times with Amicon 3 kDa cutoff filters. Finally, the resulting solution was reacted with NAD^+ (10 μL, 10 mM) in PBS buffer (50 mM, pH = 10) for 2 h at room temperature and then overnight at 4 ℃. F′-NAD^+ was obtained after washing with Amicon 3 kDa several times.

Enzyme/cofactor-DNA conjugate purification by anion-exchange FPLC

The enzyme/cofactor-DNA conjugates were diluted three times with wash buffer (20 mM phosphate buffer, pH 7.4, containing 25 mM Na^+) and were injected into the column. Then, the samples were separated by using an elution gradient from 0.25 mM Na^+ (20 mM phosphate buffer, pH 7.4) to 1 M Na^+ (20 mM phosphate buffer, pH 7.4) with a flow rate of 1.5 mL/min. The fractions of multiple peaks from the purification chromatogram were collected and analyzed by UV spectra to evaluate the ratio of enzyme and DNA.

Note that, all the molar ratio of calculation of purified enzyme/cofactor-DNA conjugates are summarized in Table S1.

Preparation of $H_{AB'}$, $H_{BA'}$, H_N, H_P, A_1B_1, A_2B_2, $H_{GH'}$, and $H_{HG'}$ hairpins

Taking $H_{AB'}$ as an example, $H_{AB'}$ (100 μM) in HEPES buffer (10 mM, pH = 7.2) that included 20 mM $MgCl_2$ was annealed at 95 °C for 5 min, cooled down quickly to 25 °C in short time and equilibrated at 25 °C for 2 h. The $H_{BA'}$, H_N, H_P, A_1B_1, and A_2B_2 hairpins follow the same procedure. GOx-modified $H_{HG'}$ and HRP-modified $H_{GH'}$ (20 uM) in 1× rCutSmart Buffer were annealed at 65 °C for 10 min, and slow cooling to 25 °C at a rate of 1 °C min^{-1}, and then allowed to equilibrate at 25 °C for 2 h. Note that, $H_{AB'}$, $H_{BA'}$, A_1B_1, A_2B_2, $H_{GH'}$, and $H_{HG'}$ hairpins contain o-nitrobenzyl phosphate photoresponsive units that are photodeprotected at λ = 365 nm.

Preparation of CDN K, CDN J, CDN Ga, and CDN Gb

Taking CDN K as an example, a mixture of C, D, C′, and D′, 2 μM each, in HEPES buffer (10 mM, pH = 7.2), which included 20 mM $MgCl_2$, was annealed at 65 °C for 10 min, and slow cooling to 25 °C at a rate of 0.3 °C min^{-1}, and then allowed to equilibrate at 25 °C for 2 h.

Light-emerged of CDN X and assessment of the concentration of each constituent in CDN X upon the Light activation.

A mixture solution of annealed $H_{AB'}$, $H_{BA'}$ hairpins containing each component of 1 μM, was irradiated under UV light (λ = 365 nm) for different time-intervals, and then incubated at 37 °C. Subsequently, aliquots of 30 μL were withdrawn from the solution at different time-intervals and treated with sub1, sub2-noFQ, sub3-noFQ, and sub4-noFQ (for AA′), or with sub1-noFQ, sub2, sub3-noFQ, and sub4-noFQ (for BB′), or with sub1-noFQ, sub2-noFQ, sub3, and sub4-noFQ (for BA′), or with sub1-noFQ, sub2-noFQ, sub3-noFQ, and sub4 (for AB′), 1.5 μL of 100 μM each substrate. The time-dependent fluorescence changes were followed at 25 °C (catalytic rate = $d(\Delta F)/dt$). By using the appropriate calibration curves corresponding to the cleavage rates of the different substrates by the intact constituents at various concentrations, the concentrations of the constituents in light-evolved equilibrated CDN X at different time

intervals of irradiation were quantified. It should be noted that the low-power light source to uncage the o-nitrobenzyl phosphate ester-locked strand did not lead to any electrophoretically detectable side products of the nucleic acid strands.

Light-evolved CDN X/H_{P-1}-triggered CDN K or CDN X/H_{N-1}-triggered CDN J, and light-evolved orthogonally simultaneous CDN X/H_{P-1}- and CDN X/H_{N-1}-triggered CDN K and J.

Taking one system as example, the light-responsive reaction module (800 μL) of light-activation of CDN X/H_{P-1}-triggered CDN K consists of annealed $H_{AB'}$, $H_{BA'}$, H_P (1 μM, each component) and equilibrated CDN K (each component of C, D, C′, and D′ is 1 μM). The prepared above light-responsive reaction module was irradiated under UV light (λ = 365 nm) for time-intervals of 15 minutes and 3 minutes, and then was incubated in 37 °C. Aliquots of 30 μL were withdrawn from the solution at different time-intervals and treated with sub5, sub6-noFQ, sub7-noFQ, and sub8-noFQ (for DC′), or with sub5-noFQ, sub6, sub7-noFQ, and sub8-noFQ (for CD′), or with sub5-noFQ, sub6-noFQ, sub7, and sub8-noFQ (for CC′), or with sub5-noFQ, sub6-noFQ, sub7-noFQ, and sub8 (for DD′), 1.5 μL of 100 μM each substrate. Subsequently, the time-dependent fluorescence changes were followed at 25 °C (catalytic rate = $d(\Delta F)/\mathrm{d}t$). By using the appropriate calibration curves corresponding to the cleavage rates of the different substrates by the intact constituents at various concentrations, the concentrations of the constituents in light-evolved CDN X/H_{P-1}-triggered CDN K different time intervals were quantified. Note that, the experimental process of probing the light-evolved CDN X/H_{N-1}-triggered reconfiguration of CDN J, was followed by the similar method. Light-evolved orthogonally simultaneous CDN X/H_{P-1}- and CDN X/H_{N-1}-triggered CDNs K and J were implemented with mixture solution of annealed $H_{AB'}$, $H_{BA'}$, H_P, H_N (1 μM, each component) and equilibrated CDN K and CDN J (each component of C, D, C′, D′, E, E′, F, F′ is 1 μM) and were followed by the similar experimental steps.

GOx/HRP cascade and LDH/NAD^+ cascade in light-evolved orthogonally simultaneous CDN X/H_{P-1}- and CDN X/H_{N-1}-triggered CDN K and J.

To follow the GOx/HRP and LDH/NAD^+ biocatalytic cascade, the orthogonally light-triggered reaction module (800 μL, each component 1 μM except H_P and H_N, which are 5 μM) was irradiated (λ = 365 nm) for 15 minutes and 3 minutes, and allowed to equilibrate at 37 °C. For GOx/HRP cascade, aliquots of 60 μL were withdrawn from the above light-triggered orthogonal reaction module solution at different time-intervals and treated with glucose (1 μL, 2.4 mM) and $ABTS^{2-}$ (1 μL, 6 mM). The absorbance changes at 420 nm were intermediately measured (probing for 2 min). For LDH/NAD^+ biocatalytic cascade, subjecting the aliquots (60 μL) of the above light-triggered orthogonal reaction module solution to 1 mM lactic acid, 1 mM hydrazine, and 50 μM MB^+ with different time intervals allows us to probe the LDH/NAD^+ cascade by the time-dependent absorbance of reduction of MB^+ at λ = 664 nm (probing for 2 min). It

should be noted that kinetic assays of the enzymes GOx, HRP, LDH and the cofactor NAD^+ did not show any degradation in their activities upon irradiation with the low-power light source, λ = 365 nm, for 30 minutes. The added volume of substrate solution (2 μL) to the analyzed, dynamically-equilibrated the reaction mixture (60 μL) has a negligible dilution effect on the CDNs constituent equilibration.

Guided modulation of thrombin catalytic activity for light-activated reaction module of CDN Ga and CDN Gb mixture by alternative auxiliary hairpin H_P or H_N

A mixture of equilibrated CDN Ga and CDN Gb (each component is 1 μM), and o-nitrobenzyl phosphate photoresponsive units-caged hairpin H_P or H_N (final concentration is 5 μM) were irradiated under UV light (λ = 365 nm) for time-interval of 15 minutes, and allowed to equilibrate at 37 °C. To follow the temporal catalytic properties of the thrombin in CDN system, aliquots of 100 μL were withdrawn from the reaction module solution at different time-intervals and treated with thrombin (1 μL, 1 μM) and fibrinogen (10 μL, 10 mg/mL) followed by probing the scattering intensity at λ = 650 nm (Excitation and emission: λ = 650 nm).

Light-evolved and fuel strand-triggered operation of orthogonal dissipative network guiding biocatalytic cascade

The inactive reaction module in 1× rCutSmart Buffer consisting of photoresponsive unit-caged hairpins $H_{HG'}$, $H_{GH'}$, A_1B_1, A_2B_2, and a nicking enzyme Nt.BbvCI, where $H_{HG'}$ modified with GOx and $H_{GH'}$ modified with HRP, was photoprotected by the UV light (λ = 365 nm) for irradiation of 15 minutes. Note that, the concentration of each component of above reaction module is 1 μM, and finial concentration of the nicking enzyme is 0.046 μM. Subsequently, the transient orthogonal CDN Y associated with two enzymes was formed and incubate for 30 minutes under 37 °C. To follow the GOx/HRP biocatalytic cascade, the activated "rest" CDN Y (400 μL, each component 1 μM) was subjected to the fuel strand A_1' or A_2' (6 μM), and allowed to equilibrate at 37 °C. Aliquots of 60 μL were withdrawn from the above A_1'- or A_2'-triggered CDN Y solution at different time-intervals and treated with glucose (1 μL, 2.4 mM) and $ABTS^{2-}$ (1 μL, 6 mM). The absorbance changes at 420 nm were intermediately measured (probing for 2 min).

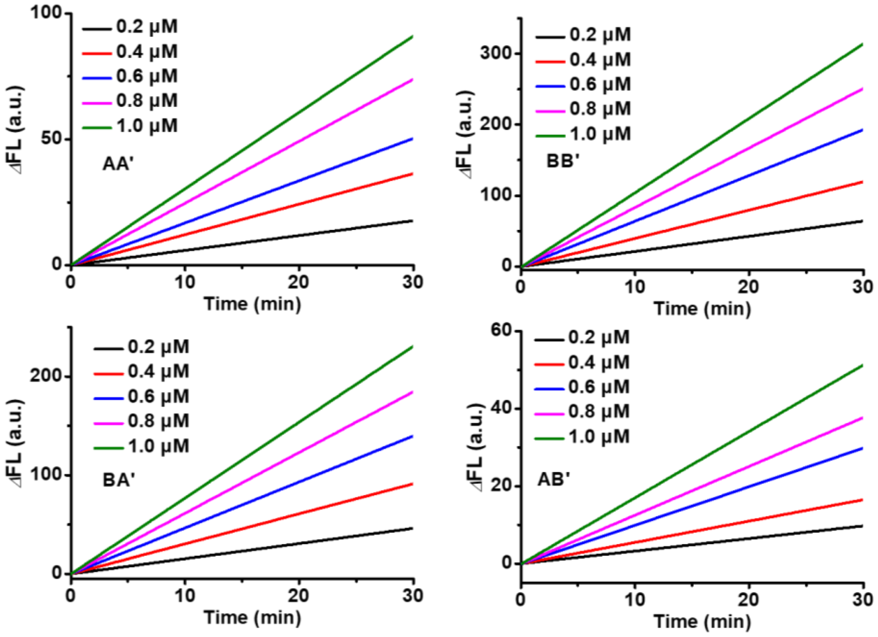

Figure S1. Time-dependent fluorescence changes generated from the cleavage of the fluorophore (F_i)/quencher (Q_i)-modified substrates by the respective DNAzyme reporter units associated with the individual constituents (of CDN X) at variable concentrations.

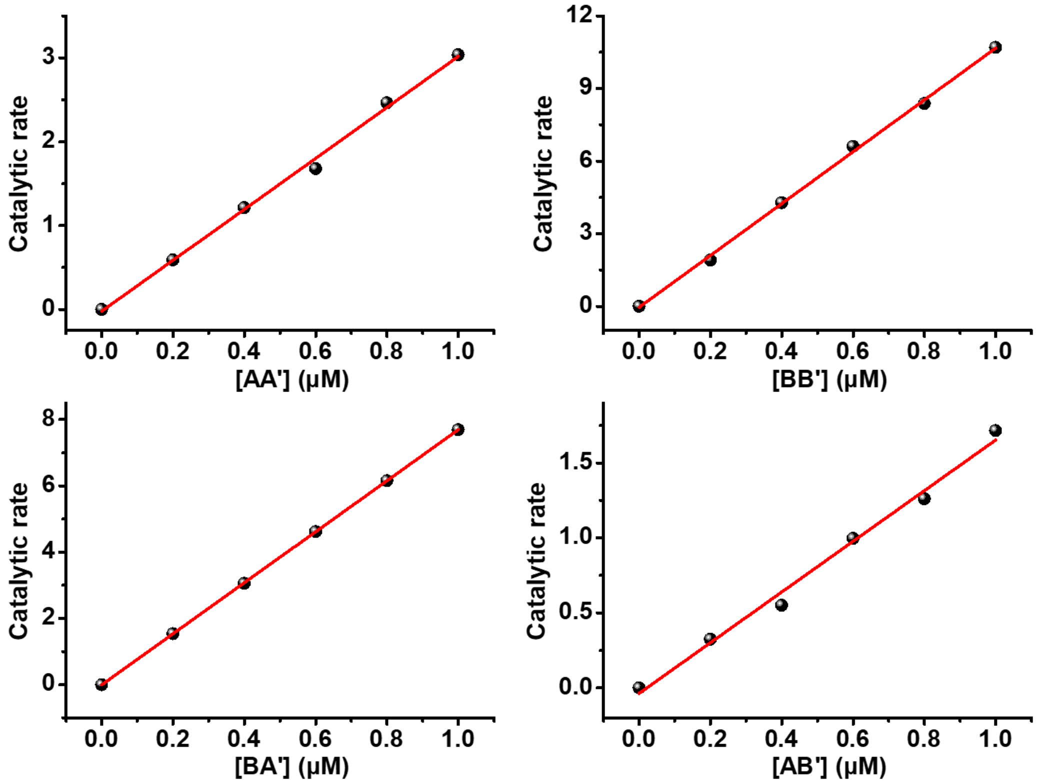

Figure S2. Calibration curves of constituents in CDN X as a function of their concentrations.

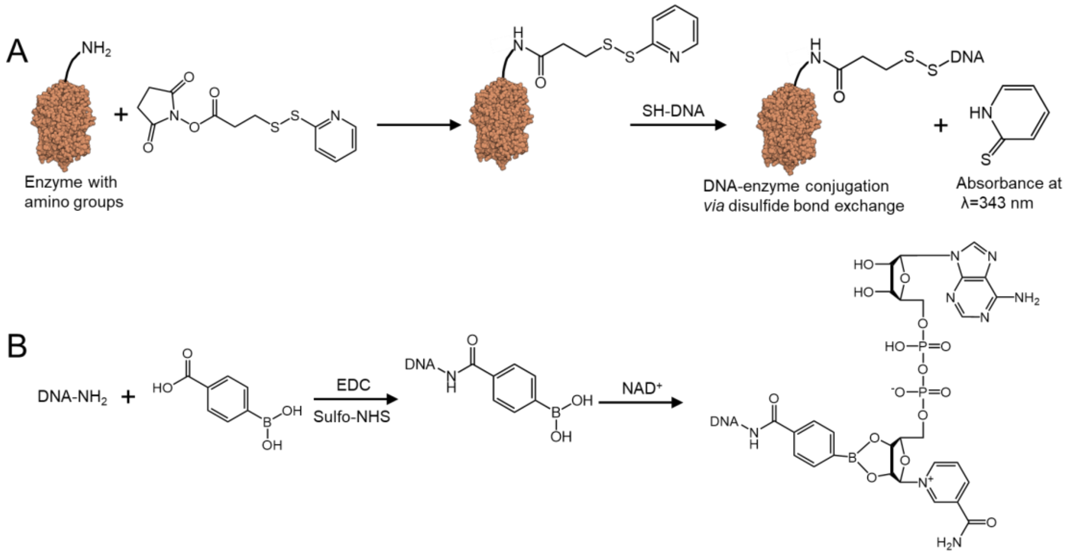

Figure S3. (A) Synthetic route on enzymes (GOx, HRP, LDH)-DNA conjugates. (B) Synthetic route of NAD$^+$-DNA conjugates.

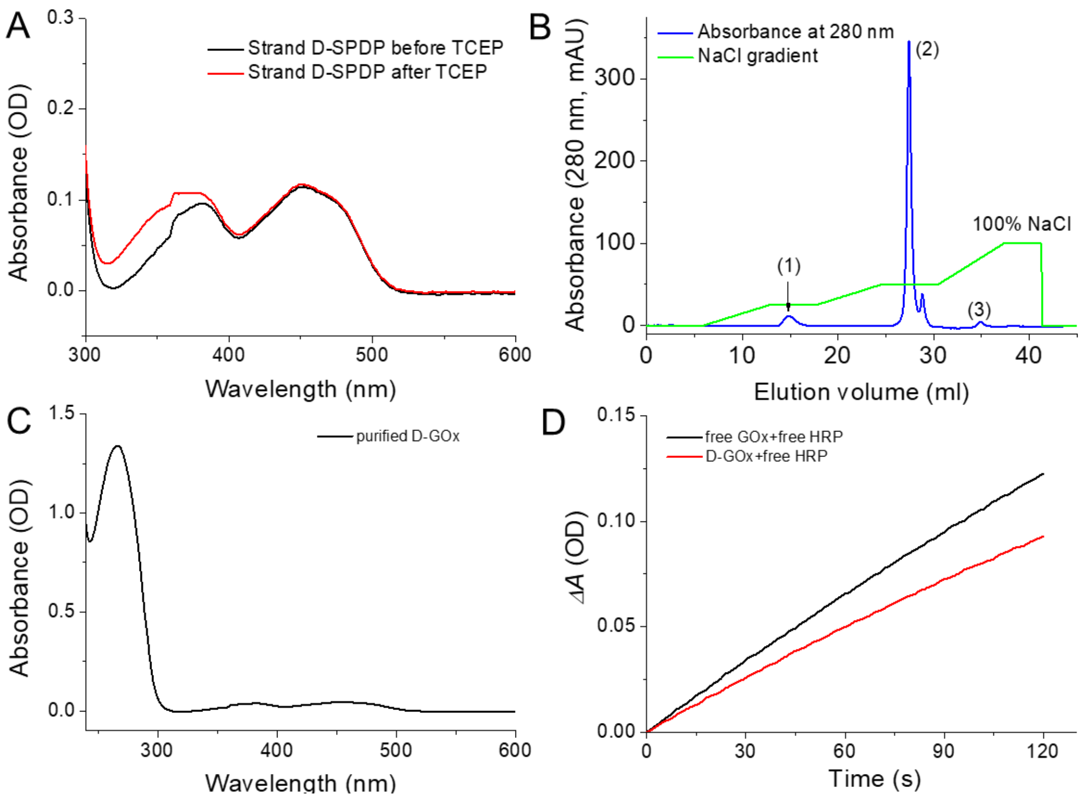

Figure S4. (A) Absorbance spectra of GOx-SPDP before and after the treatment of D: ΔA (λ = 343 nm) before and after the treatment of TCEP is ~ 0.044 (extinction coefficient of pyridine-2-thione: 8,080 M^{-1} cm^{-1}), corresponding to 5.4 μM SPDP coupled with 5.1 μM GOx (extinction coefficient of GOx at 450 nm is 22600 M^{-1} cm^{-1}). TCEP is used to calculate the coupling efficiency between GOx and SPDP due to the release of pyridine-2-thione. (B) Anion-exchange FPLC to purify DNA-conjugated GOx. The GOx and GOx modified with different strand D numbers are separated into distinct peaks that are collected in fractions: peak (1)-unmodified GOx; peak (2)-GOx labelled with one strand D; peak (3)-unlabeled strand D. (C) The absorbance spectrum of purified D-GOx conjugate collected from peak (2) in anion-exchange FPLC spectrum. (D) Comparison of activity of D-GOx and native GOx.

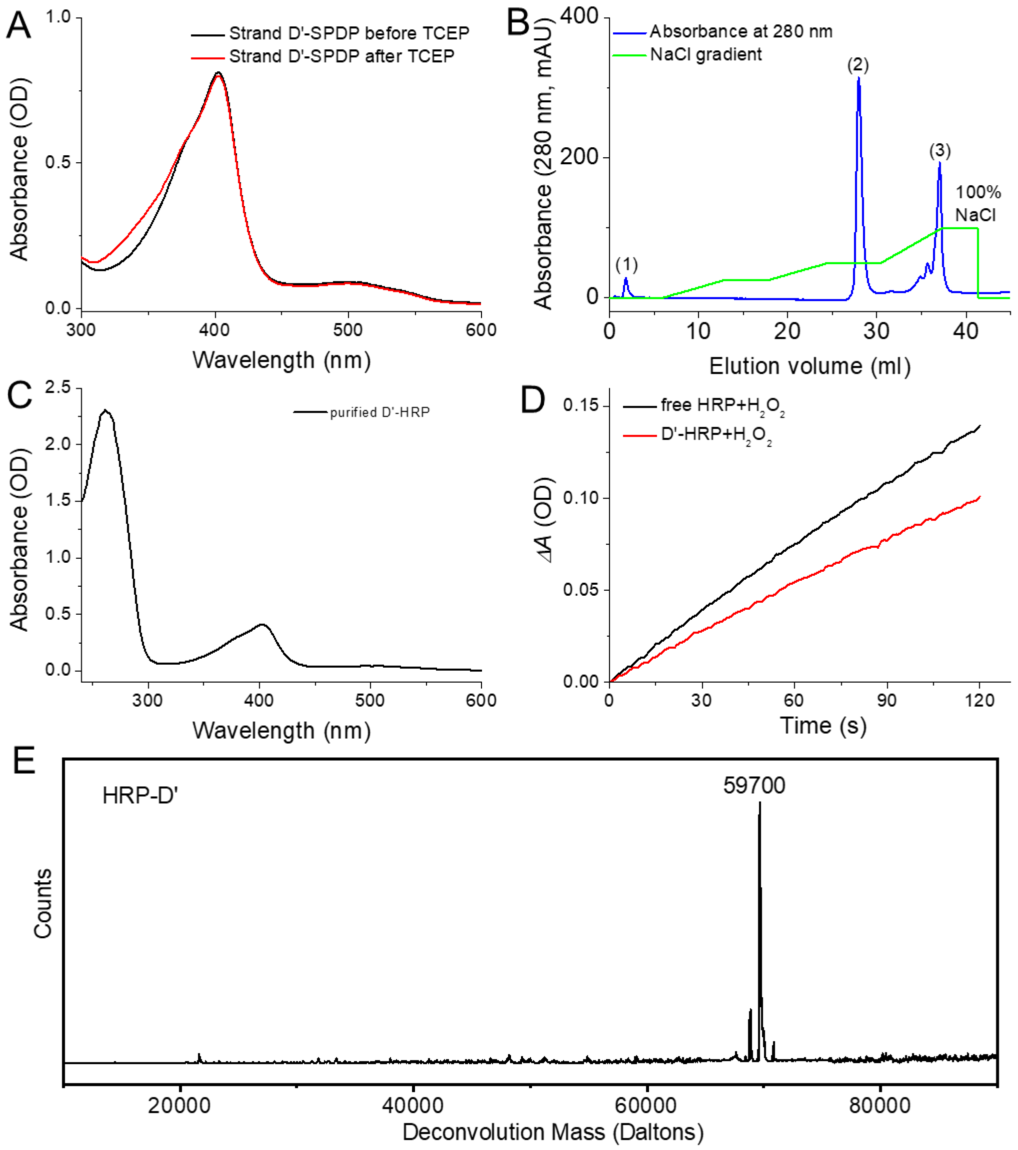

Figure S5. (A) Absorbance spectra of HRP-SPDP before and after the treatment of strand D′: ΔA (λ = 343 nm) before and after the treatment of TCEP is ~ 0.076 (extinction coefficient of pyridine-2-thione: 8,080 M^{-1} cm^{-1}), corresponding to 9.4 µM SPDP coupled with 8.03 µM HRP (extinction coefficient of HRP at 405 nm is 100000 M^{-1} cm^{-1}). TCEP is used to calculate the coupling efficiency between HRP and SPDP due to the release of pyridine-2-thione. (B) Anion-exchange FPLC to purify DNA-conjugated HRP. The HRP and HRP modified with different strand D′ number are separated into distinct peaks that are collected in fractions: peak (1)-unmodified HRP; peak (2)-HRP labelled with one strand B; peak (3)-unlabeled strand D′. (C) The absorbance spectrum of purified B′-GOx conjugate collected from peak (2) in anion-exchange FPLC spectrum. (D) Comparison of activity of D’-HRP and native HRP. (E) Transformed mass spectra by deconvolution of purified conjugate HRP-D′.

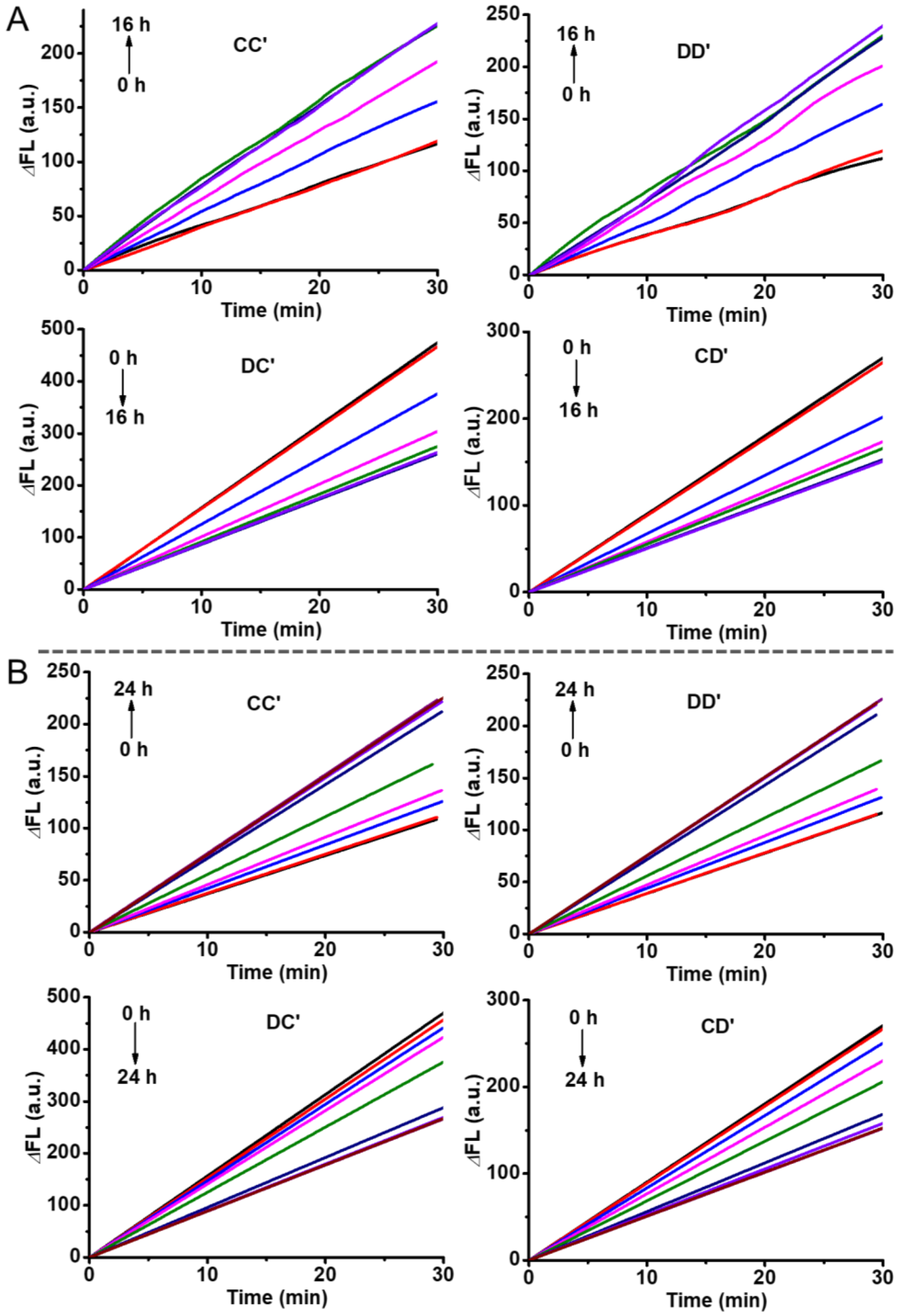

Figure S6. (A) Time-dependent fluorescence changes of Q_i/F_i-modified substrate cleaved by the DNAzyme reporter units at different time intervals during the CDN X/H_{P-1}-triggered reconfiguration of CDN K, upon irradiating the reaction module for time-interval of 15 min (A) and 3 minutes (B).

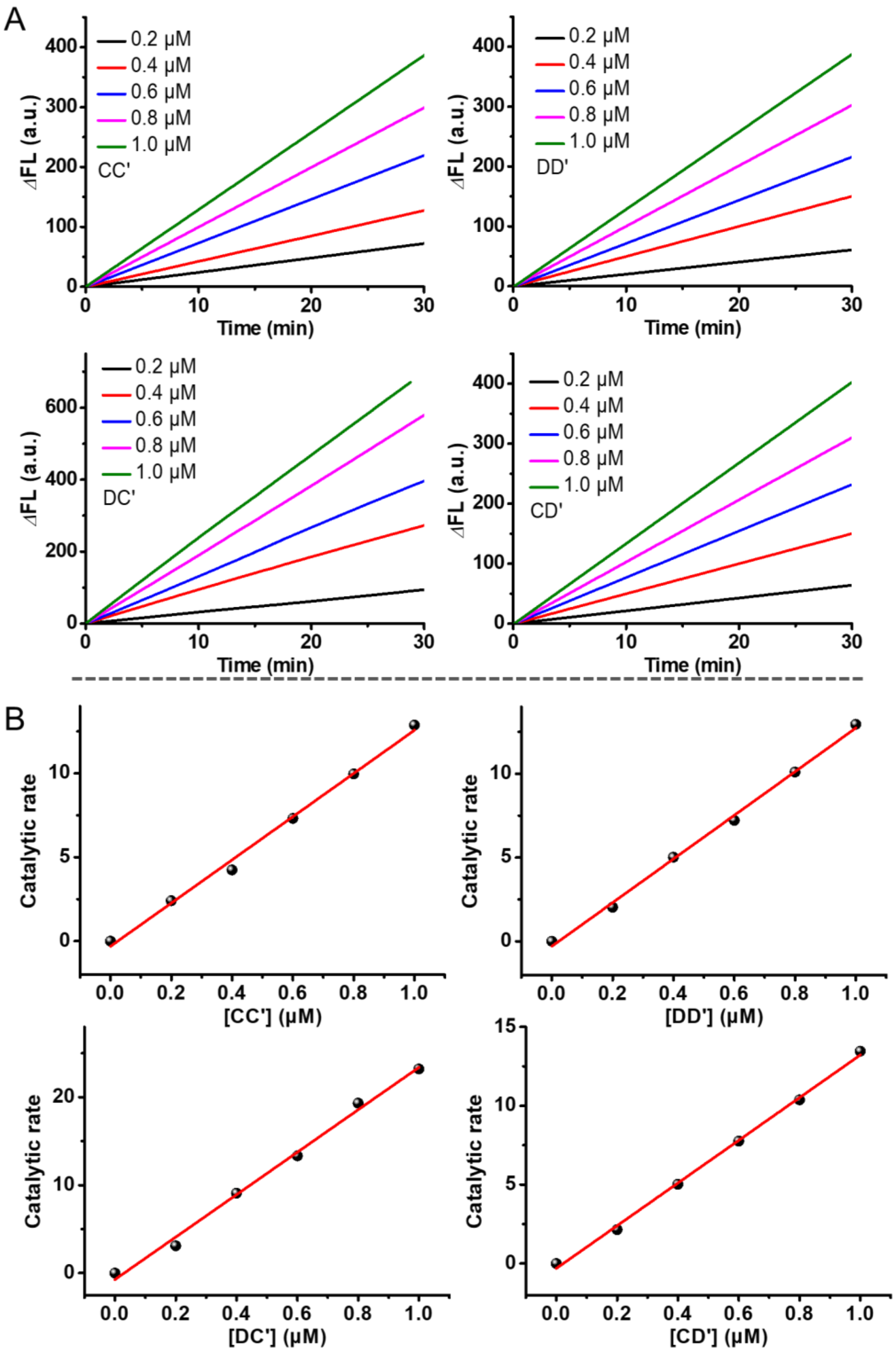

Figure S7. (A) Time-dependent fluorescence changes generated from the cleavage of the fluorophore (F_i)/quencher (Q_i)-modified substrates by the respective DNAzyme reporter units associated with the individual constituents (of CDN K) at variable concentrations. (B) Calibration curves of constituents in CDN K as a function of their concentrations.

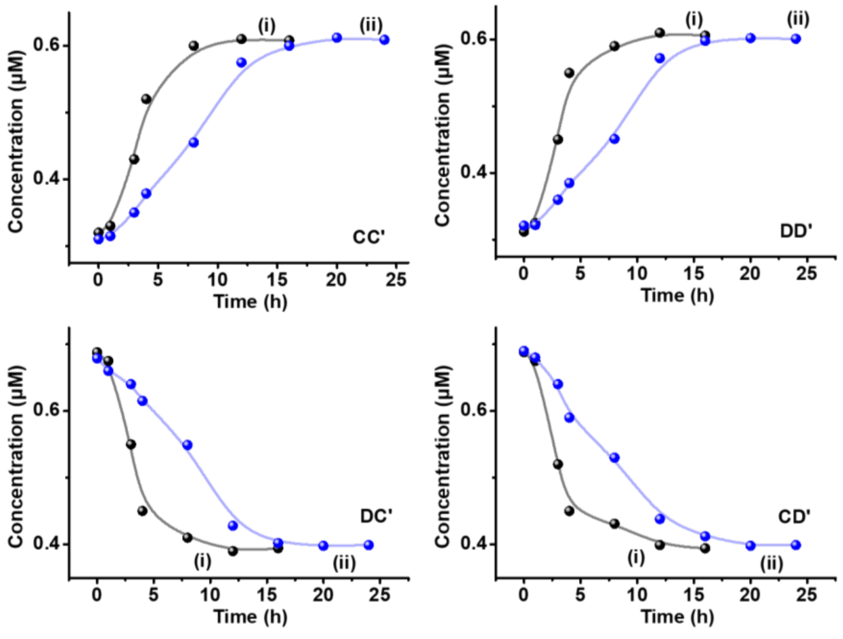

Figure S8. Dynamic concentration changes of four constituents associated with CDN K activated by CDN X/H_{P-1} upon irradiating the reaction module for time-intervals of: curves (i), 15 minutes; curves (ii) 3 minutes.

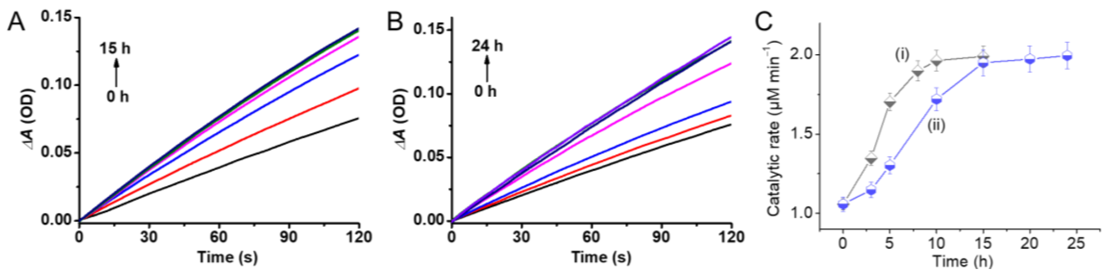

Figure S9. (A) Time-dependent absorbance change generated by the GOx/HRP biocatalytic cascade (cf. Figure 2A, Panel I) formed by samples withdrawn from the CDN X/$H_{P\text{-}1}$-fueled activation of CDN K at time-intervals of 0-15 hours. (B) Time-dependent absorbance change generated by the GOx/HRP biocatalytic cascade (cf. Figure 2A, Panel I) formed by samples withdrawn from the CDN X/$H_{P\text{-}1}$-fueled activation of CDN K at time-intervals of 0-24 hours. (C) Temporal catalytic rates corresponding to the GOx/HRP cascade driven by the CDN X/$H_{P\text{-}1}$-fueled activation of CDN K, upon the irradiation of the reaction module for time-intervals of: curve (i), 15 minutes; curve (ii), 3 minutes.

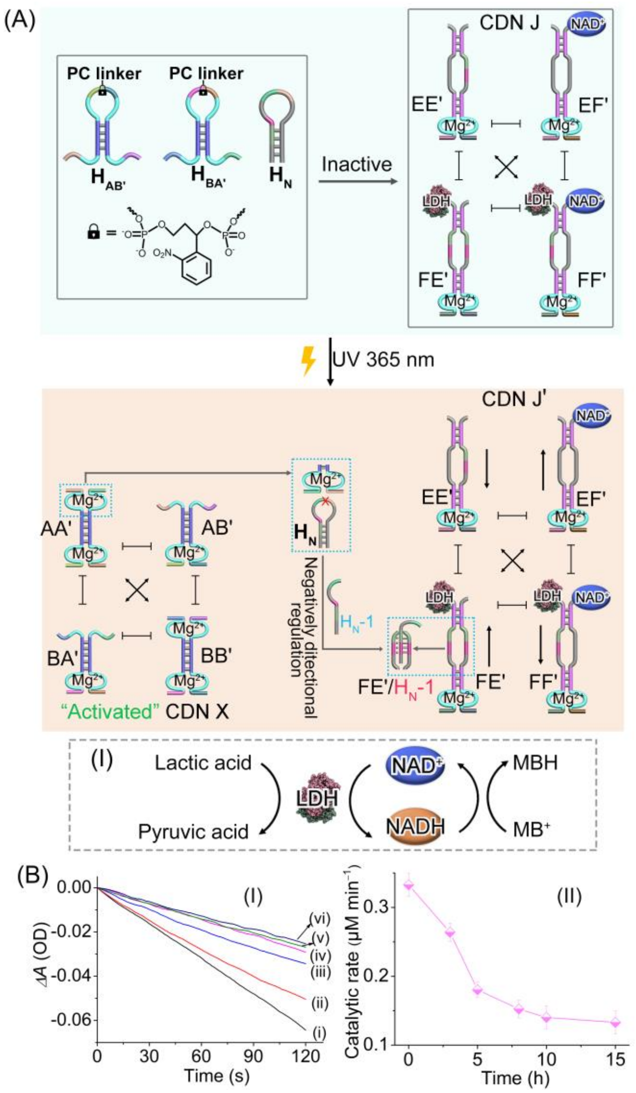

Figure S10. (A) Schematic light-triggered interaction between a reaction module consisting of photocleavable caged hairpins $H_{AB'}$, $H_{BA'}$ and fuel hairpin H_N and an auxiliary CDN J operating the LDH/NAD$^+$ biocatalytic cascade. Phototriggered cleavage of the reaction module evolves CDN X that cleaves the hairpin H_N. The cleaved fuel strand H_{N-1} stabilizes constituent FE′ of auxiliary CDN J, resulting in the downregulation of the LDH/NAD$^+$ biocatalytic cascade (detailed in Panel I). (B) Panel I-Time-dependent absorbance change generated by the LDH/NAD$^+$ biocatalytic cascade (cf. Figure S10A, Panel I) formed by samples withdrawn at time-intervals from the CDN X/H_{N-1}-fueled operating CDN J: (i) 0, (ii) 3, (iii) 5, (iv) 8, (v) 10, (vi) 15 hours.

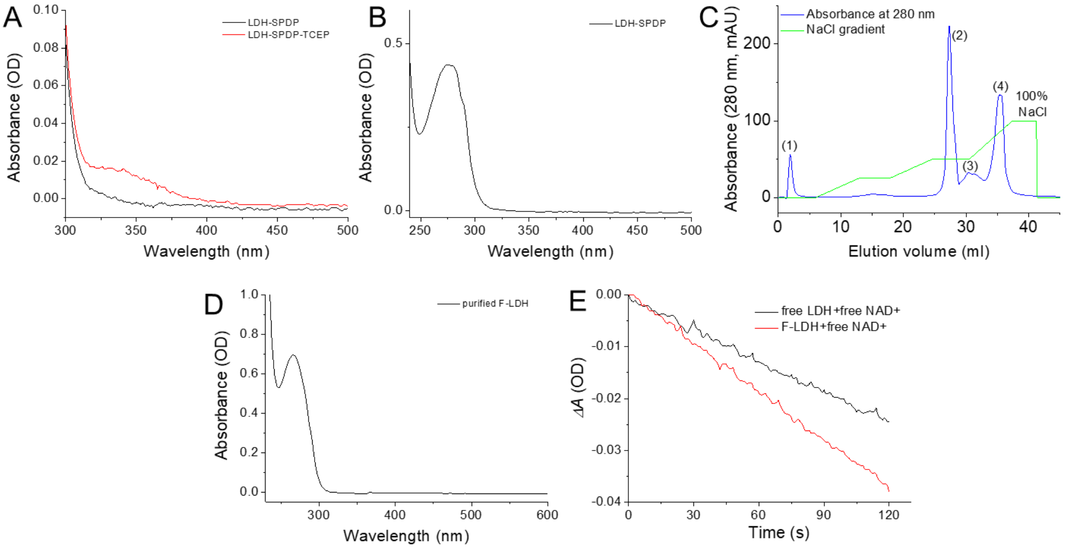

Figure S11. (A-B) Absorbance spectra of LDH-SPDP before and after the treatment of TCEP: ΔA (λ = 343 nm) before and after the treatment of TCEP is ~0.0153 (extinction coefficient of pyridine-2-thione: 8,080 M^{-1} cm^{-1}), corresponding to 1.9 µM SPDP coupled with 2.3 µM LDH (extinction coefficient of LDH at 280 nm is 186,502 M^{-1} cm^{-1}). TCEP is used to calculate the coupling efficiency between LDH and SPDP due to the release of pyridine-2-thione. (C) Anion-exchange FPLC to purify DNA-conjugated LDH. The LDH and LDH modified with different strand F number are separated into distinct peaks that are collected in fractions: peak (1)-unmodified LDH; peak (2)-LDH labelled with one strand F; peak (4)-unlabeled strand F. (D) The absorbance spectrum of purified LDH-F conjugate collected from peak (2) in anion-exchange FPLC spectrum. (E) Comparison of activity of F-LDH and native LDH.

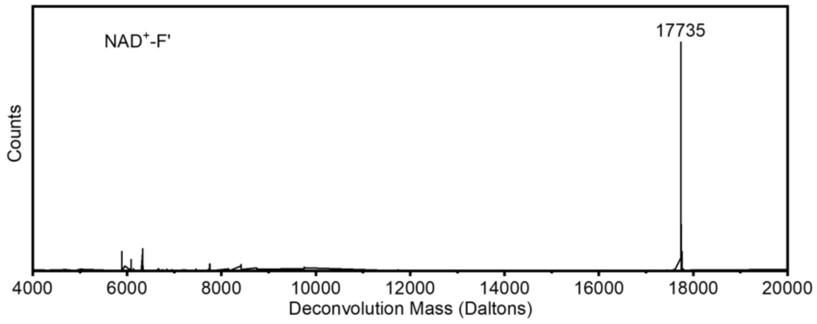

Figure S12. Transformed mass spectrum by deconvolution of conjugate NAD^{+}-F'.

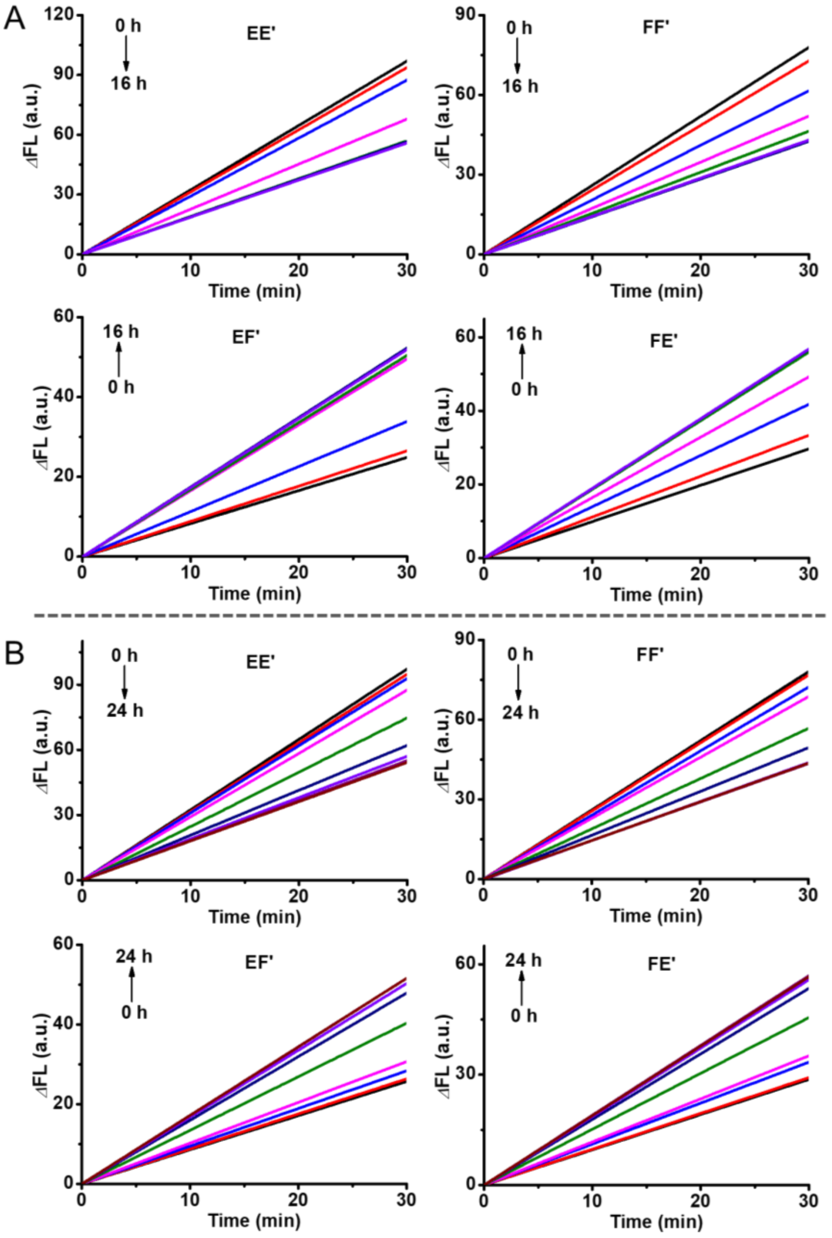

Figure S13. (A) Time-dependent fluorescence changes of Q_i/F_i-modified substrate cleaved by the DNAzyme reporter units at different time intervals during the CDN X/H_{N-1}-triggered reconfiguration of CDN J, upon irradiating the reaction module for time-interval of 15 min (A) and 3 minutes (B).

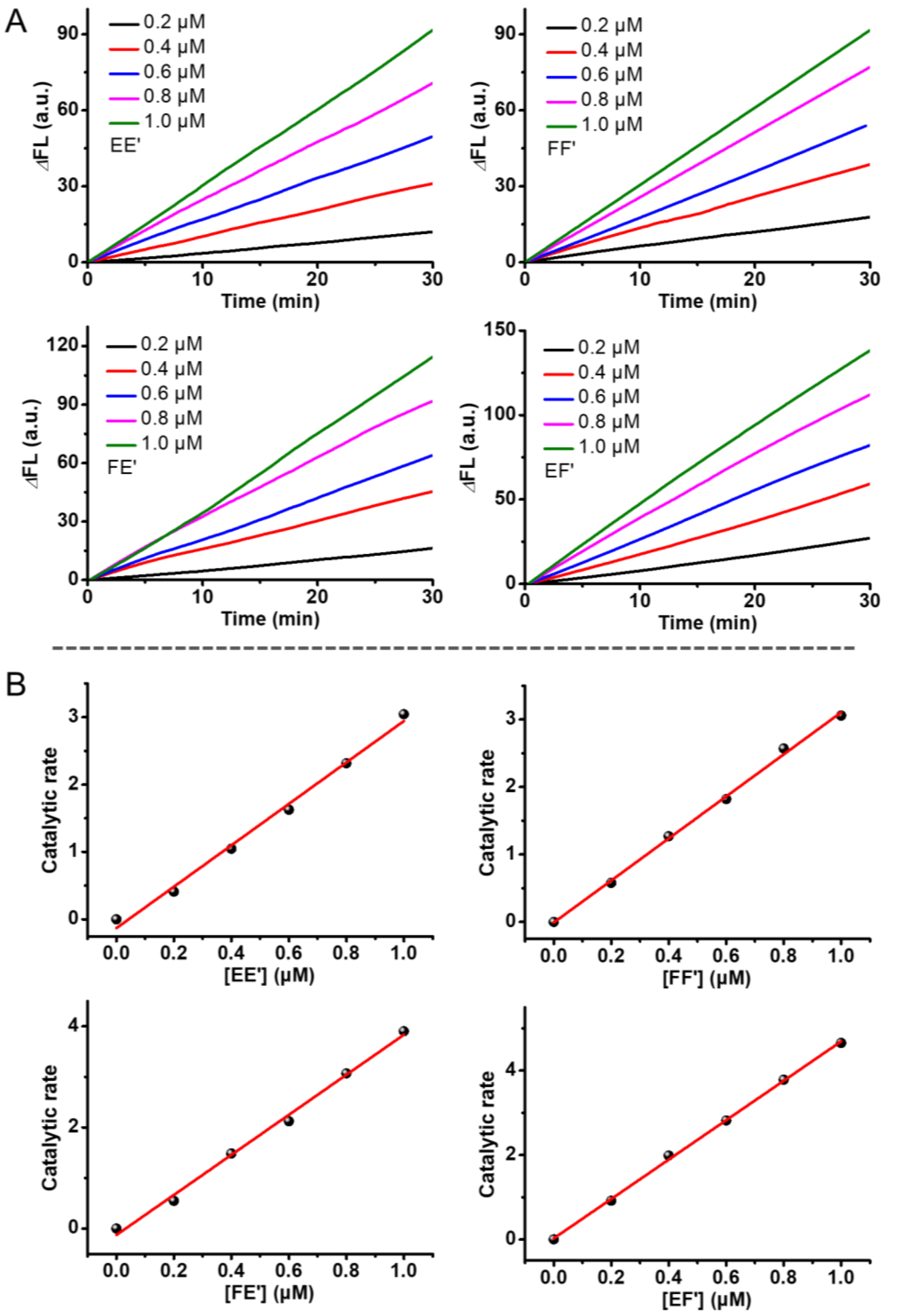

Figure S14. (A) Time-dependent fluorescence changes generated from the cleavage of the fluorophore (F_i)/quencher (Q_i)-modified substrates by the respective DNAzyme reporter units associated with the individual constituents (of CDN J) at variable concentrations. (B) Calibration curves of constituents in CDN J as a function of their concentrations.

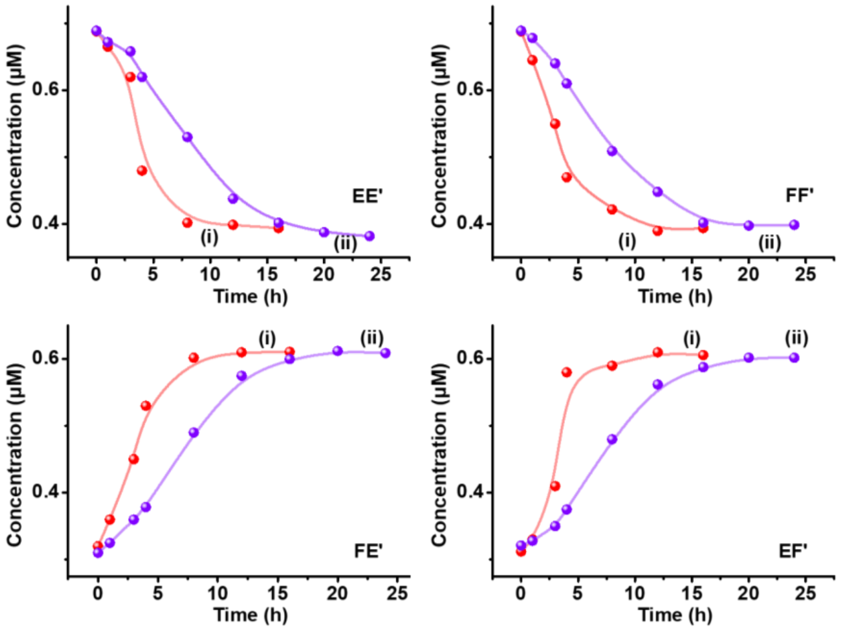

Figure S15. Dynamic concentration changes of four constituents associated with CDN J activated by CDN X/H_{N-1} upon irradiating the reaction module for time-intervals of: curves (i), 15 minutes; curves (ii) 3 minutes.

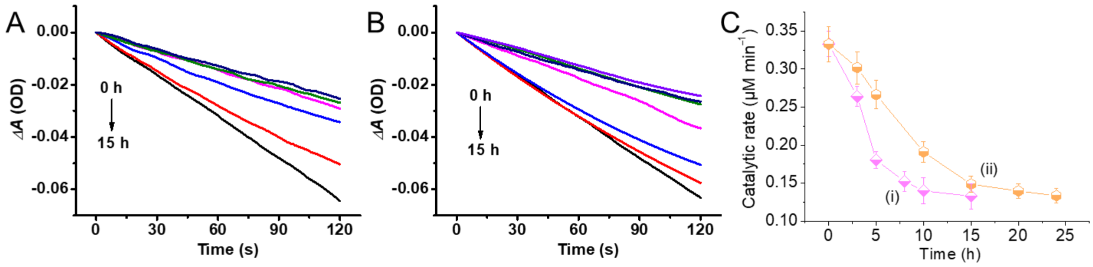

Figure S16. (A) Time-dependent absorbance change generated by the LDH/NAD$^+$ biocatalytic cascade (cf. Figure 2C, Panel I) formed by samples withdrawn from the CDN X/H$_{N-1}$-fueled activation of CDN J at time-intervals of 0-15 hours. (B) Time-dependent absorbance change generated by the LDH/NAD$^+$ biocatalytic cascade formed by samples withdrawn from the CDN X/H$_{N-1}$-fueled activation of CDN J at time-intervals of 0-24 hours. (C) Temporal catalytic rates corresponding to the LDH/NAD$^+$ cascade driven by the CDN X/H$_{N-1}$-fueled activation of CDN J, upon the irradiation of the reaction module for time-intervals of: curve (i), 15 minutes; curve (ii), 3 minutes.

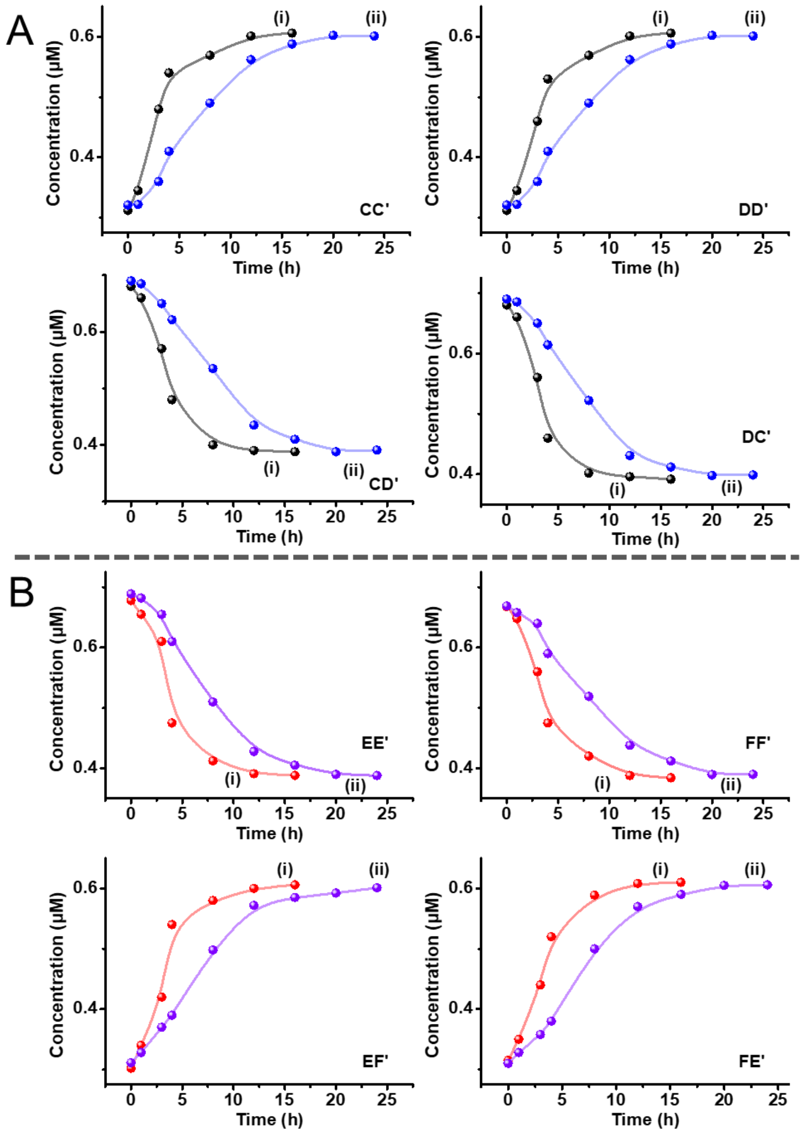

Figure S17. (A) Dynamic concentration changes of four constituents associated with CDN K activated by CDN X/H_{P-1} and (B) Dynamic concentration changes of four constituents associated with CDN J activated by CDN X/H_{N-1}, upon irradiating the orthogonal reaction module for time-intervals of: curves (i), 15 minutes; curves (ii) 3 minutes.

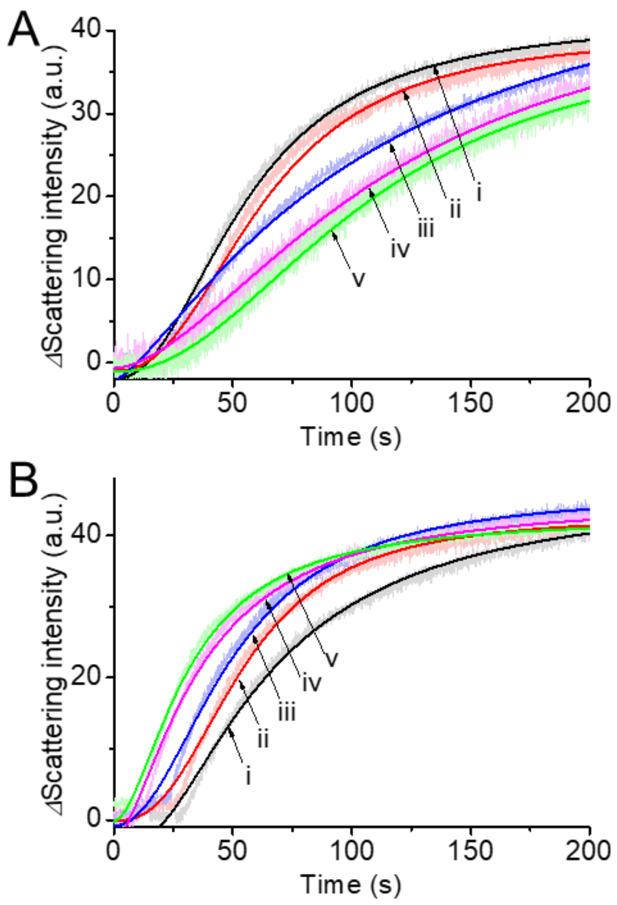

Figure S18. (A) Temporal light-scattering intensity changes corresponding to the temporal coagulation of fibrinogen to fibrin upon the light-triggered CDN X/H_{P-1}-guided upregulation of thrombin-aptamer-modified LL′ in CDN Ga. Light-scattering curves are recorded on samples withdrawn from the system at time intervals corresponding to (i) 0, (ii) 3, (iii) 5, (iv) 10, and (v) 15 hours. (B) Temporal light-scattering intensity changes corresponding to the temporal coagulation of fibrinogen to fibrin upon the light-triggered CDN X/H_{N-1}-guided downregulation of thrombin-aptamer-modified QQ′ in CDN Gb. Light-scattering curves are recorded on samples withdrawn from the system at time intervals corresponding to (i) 0, (ii) 3, (iii) 5, (iv) 10, and (v) 15 hours.

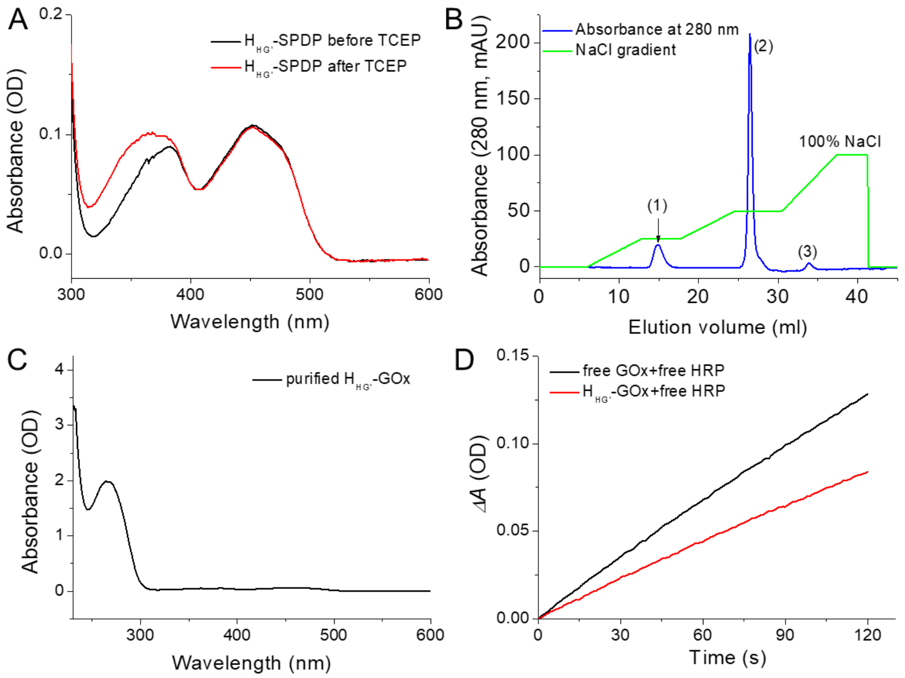

Figure S19. (A) Absorbance spectra of GOx-SPDP before and after the treatment of $H_{HG'}$: ΔA (λ = 343 nm) before and after the treatment of TCEP is ~ 0.038 (extinction coefficient of pyridine-2-thione: 8,080 M^{-1} cm^{-1}), corresponding to 4.7 µM SPDP coupled with 4.6 µM GOx (extinction coefficient of GOx at 450 nm is 22600 M^{-1} cm^{-1}). TCEP is used to calculate the coupling efficiency between GOx and SPDP due to the release of pyridine-2-thione. (B) Anion-exchange FPLC to purify DNA-conjugated GOx. The GOx and GOx modified with different strand $H_{HG'}$ numbers are separated into distinct peaks that are collected in fractions: peak (1)-unmodified GOx; peak (2)-GOx labelled with one strand $H_{HG'}$; peak (3)-unlabeled strand $H_{HG'}$. (C) The absorbance spectrum of purified $H_{HG'}$-GOx conjugate collected from peak (2) in anion-exchange FPLC spectrum. (D) Comparison of activity of $H_{HG'}$-GOx and native GOx.

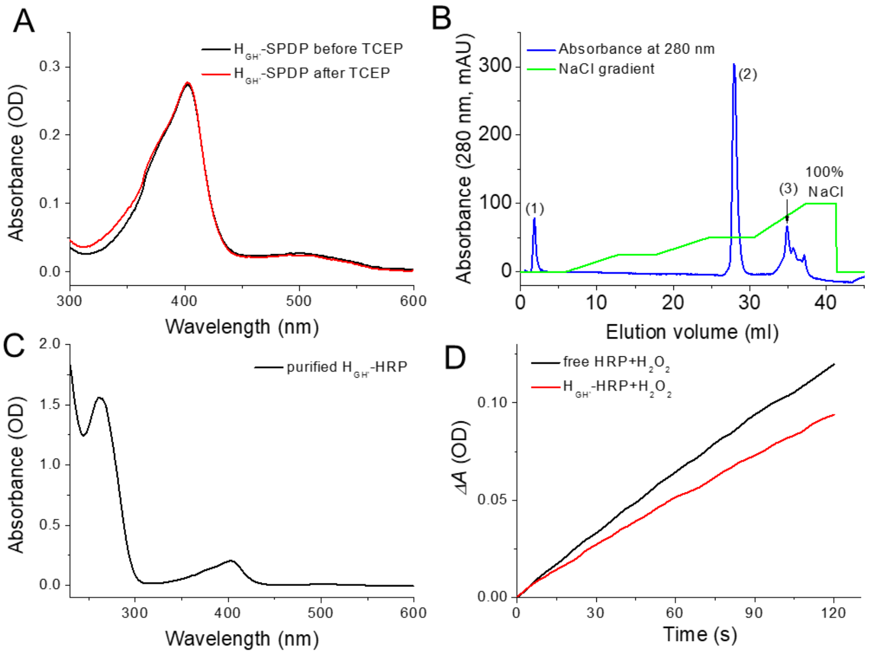

Figure S20. (A) Absorbance spectra of HRP-SPDP before and after the treatment of strand $H_{GH'}$: ΔA (λ = 343 nm) before and after the treatment of TCEP is ~ 0.0186 (extinction coefficient of pyridine-2-thione: 8,080 M^{-1} cm^{-1}), corresponding to 2.3 µM SPDP coupled with 2.7 µM HRP (extinction coefficient of HRP at 405 nm is 100000 M^{-1} cm^{-1}). TCEP is used to calculate the coupling efficiency between HRP and SPDP due to the release of pyridine-2-thione. (B) Anion-exchange FPLC to purify DNA-conjugated HRP. The HRP and HRP modified with different strand $H_{GH'}$ number are separated into distinct peaks that are collected in fractions: peak (1)-unmodified HRP; peak (2)-HRP labelled with one strand $H_{GH'}$; peak (3)-unlabeled strand $H_{GH'}$. (C) The absorbance spectrum of purified $H_{GH'}$-HRP conjugate collected from peak (2) in anion-exchange FPLC spectrum. (D) Comparison of activity of $H_{GH'}$-HRP and native HRP.

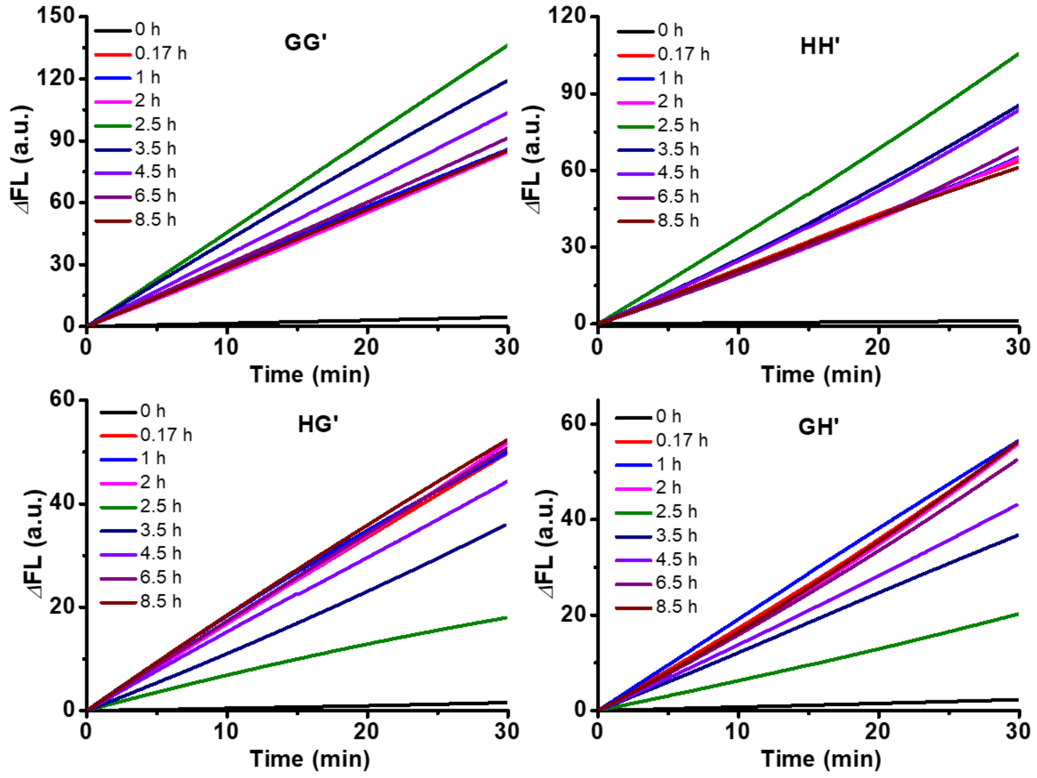

Figure S21. Time-dependent catalytic activity changes of the DNAzyme reporter units associated with the constituents at different time intervals during the dissipative transitions of CDN Y→Y1→Y, upon subjecting light-emerged transient CDN Y reaction module that includes the nicking enzyme, 0.046 μM, to the fuel strand A_1', 6.0 μM.

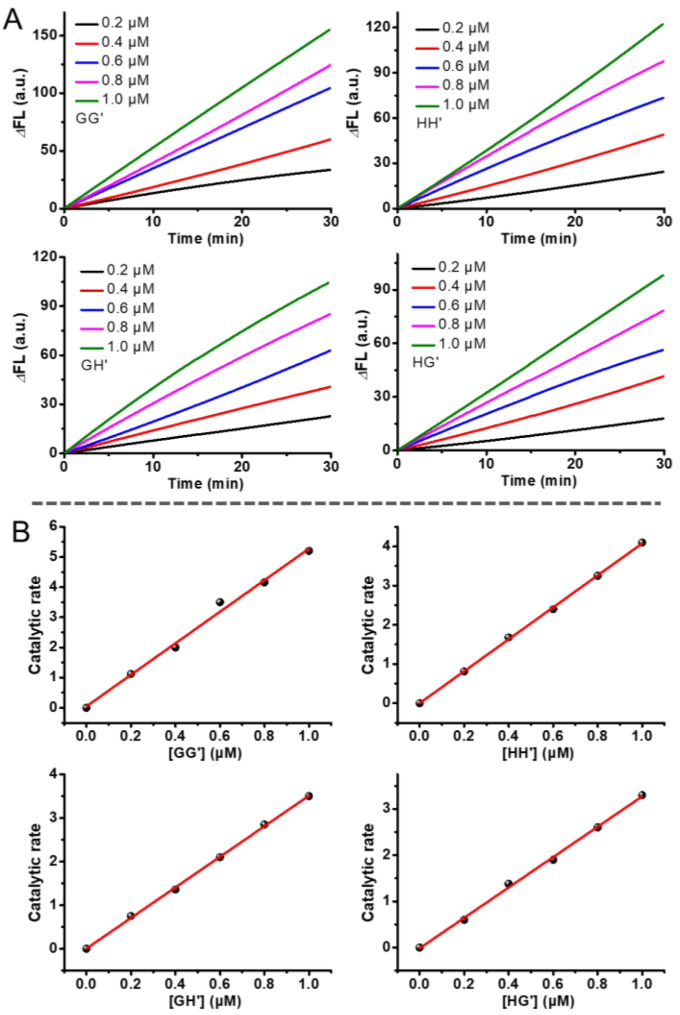

Figure S22. (A) Time-dependent fluorescence changes generated from the cleavage of the fluorophore (F_i)/quencher (Q_i)-modified substrates by the respective DNAzyme reporter units associated with the individual constituents (of CDN Y) at variable concentrations. (B) Calibration curves of constituents in CDN Y as a function of their concentrations.

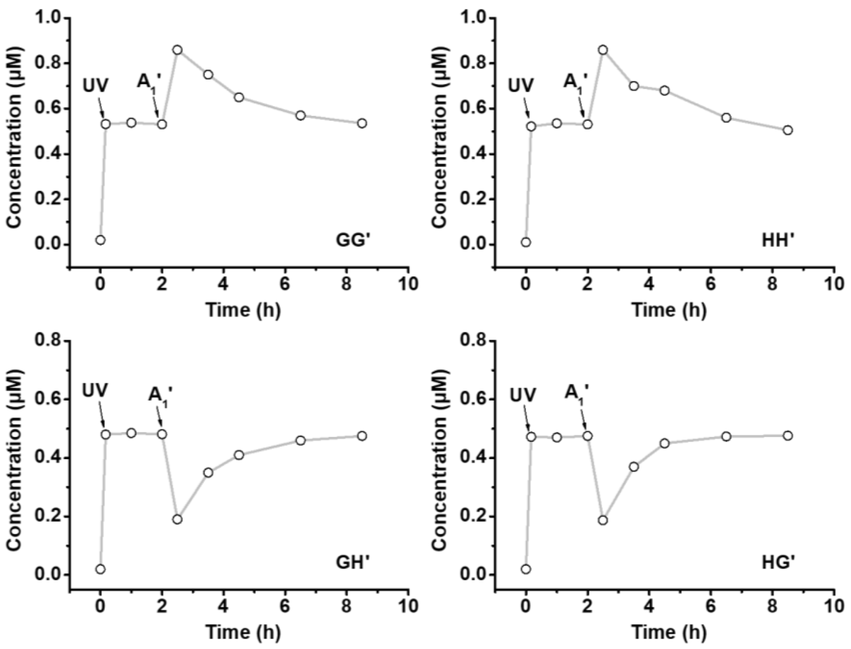

Figure S23. Dynamic concentration changes of four constituents associated with light-evolved transient CDN Y network module during the dissipative transitions of CDN Y→Y1→Y, upon subjecting light-emerged transient CDN Y reaction module that includes the nicking enzyme, 0.046 μM, to the fuel strand A_1', 6.0 μM.

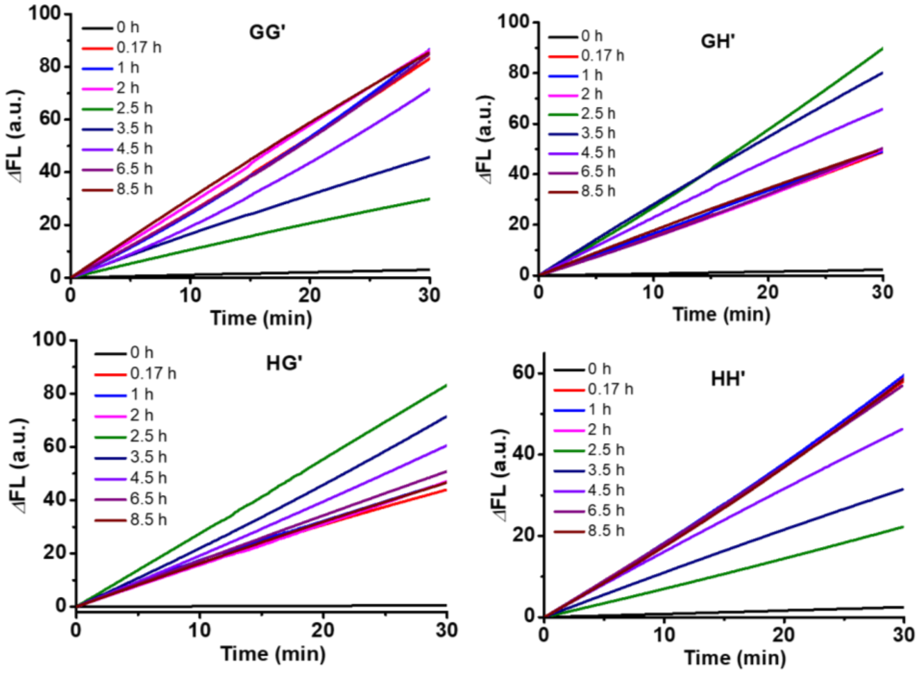

Figure S24. Time-dependent catalytic activity changes of the DNAzyme reporter units associated with the constituents at different time intervals during the dissipative transitions of CDN Y→Y2→Y, upon subjecting light-emerged transient CDN Y reaction module that includes the nicking enzyme, 0.046 μM, to the fuel strand A_2', 6.0 μM.

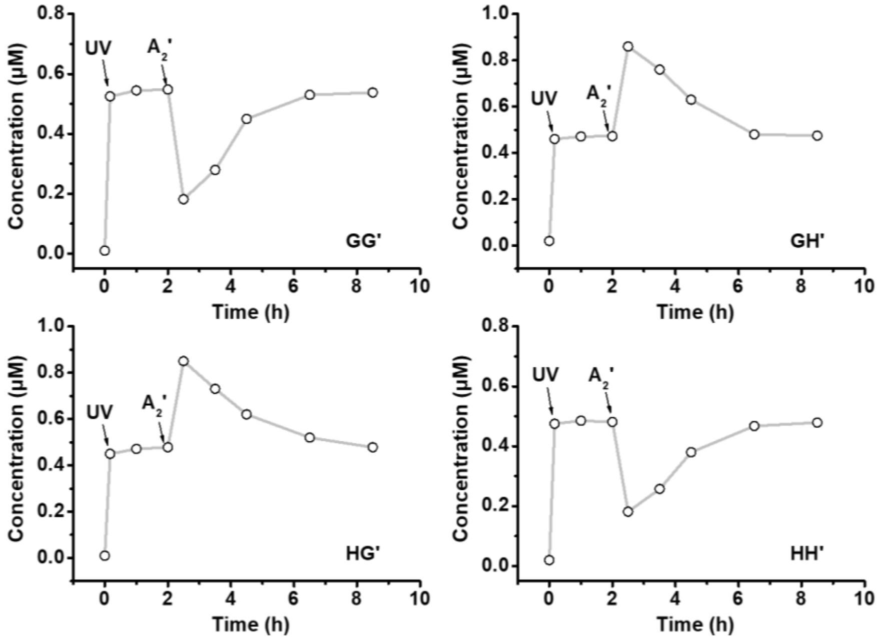

Figure S25. Dynamic concentration changes of four constituents associated with light-evolved transient CDN Y network module during the dissipative transitions of CDN Y→Y2→Y, upon subjecting light-emerged transient CDN Y reaction module that includes the nicking enzyme, 0.046 μM, to the fuel strand A_2', 6.0 μM.

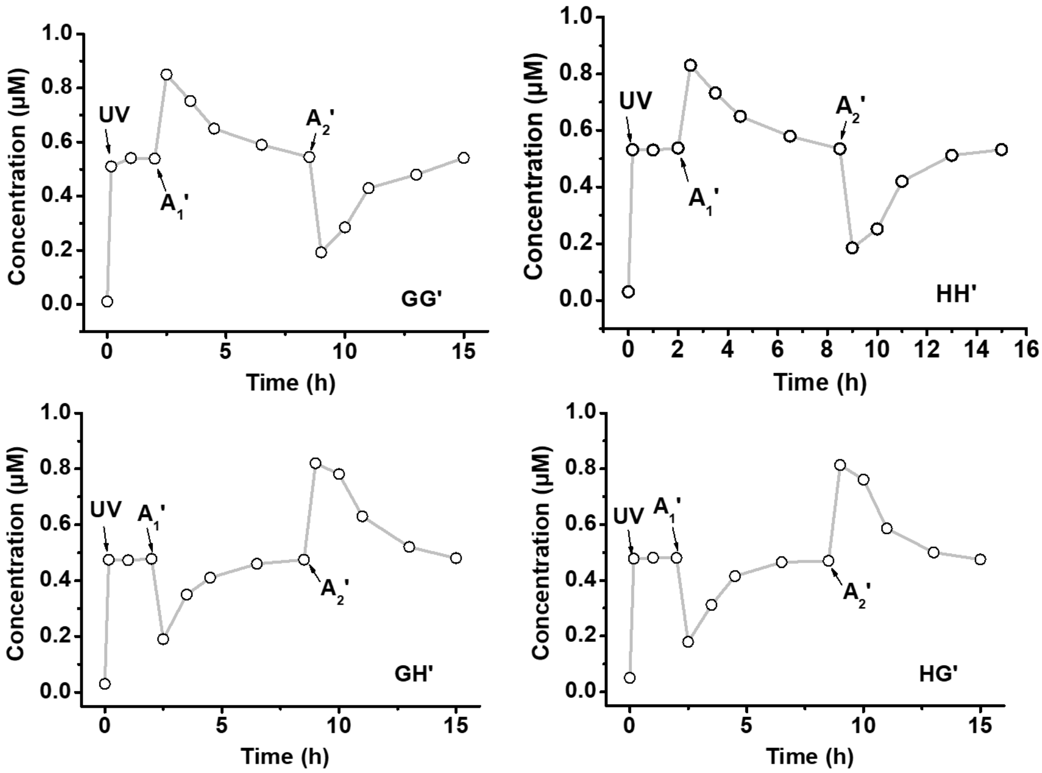

Figure S26. Dynamic concentration changes of four constituents associated with light-evolved transient CDN Y network module during the dissipative transitions of CDN Y→Y1→Y→Y2→Y, upon subjecting light-emerged transient CDN Y reaction module to the nicking enzyme, 0.046 μM, to the consecutive fuel strand A_1' and A_2', 6.0 μM.

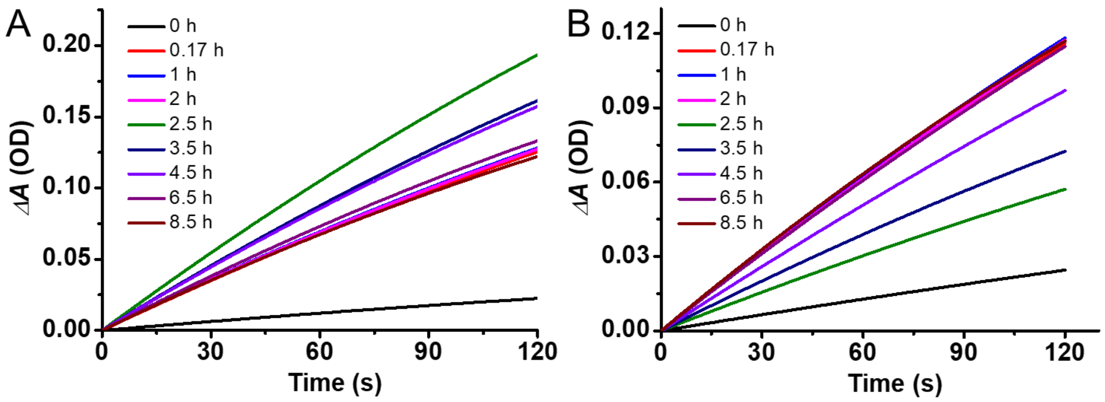

Figure S27. (A) Time-dependent absorbance changes of GOx/HRP biocatalytic cascade associated with constituent of HH′ in light-evolved transient CDN Y network module during the dissipative transitions of CDN Y→Y1→Y, upon subjecting light-emerged transient CDN Y reaction module that includes the nicking enzyme, 0.046 μM, to the fuel strand A_1', 6.0 μM. (B) Time-dependent absorbance changes of GOx/HRP biocatalytic cascade associated with constituent of HH′ in light-evolved transient CDN Y network module during the dissipative transitions of CDN Y→Y2→Y, upon subjecting light-emerged transient CDN Y reaction module that includes the nicking enzyme, 0.046 μM, to the fuel strand A_2', 6.0 μM.

Table S1. Quantification of the concentration and DNA labeling ratio of the purified enzyme-DNA conjugates by measuring the absorbance at 260 and 280 nm

**Enzyme**	**ε_{260} (M^{-1} cm^{-1})**	**ε_{280} (M^{-1} cm^{-1})**	**E_{405} (M^{-1} cm^{-1})**	**E_{450} (M^{-1} cm^{-1})**
GOx	168336	267200	-	22600
HRP	38000	-	100000	-
LDH	132175	186502	-	-
**DNA**	**ε_{260} (M^{-1} cm^{-1})**	**ε_{280} (M^{-1} cm^{-1})**		
D	500300	269207		
D′	510100	360515		
F	492100	325260		
$H_{HG'}$	756100	477536		
$H_{GH'}$	646100	368526		
**Purified DNA-Enzyme**	**Ratio$_{(DNA: Enzyme)}$**			
D-GOx	1.06:1			
D′-HRP	1.04:1			
F-LDH	1.08:1			
$H_{HG'}$-GOx	1.07:1			
$H_{GH'}$-HRP	1.09:1			

A_{260} (Enzyme-DNA) = ε_{260} (enzyme) × C (enzyme) + ε_{260} (DNA) × C (DNA) (1)

A_{280} (Enzyme-DNA) = ε_{280} (enzyme) × C (enzyme) + ε_{280} (DNA) × C (DNA) (2)

$$Ratio\left(\frac{DNA}{enzyme}\right) = \frac{c_{DNA}}{c_{enzyme}} \quad (3)$$
